# Dissecting the Reduced Penetrance of Putative Loss-of-Function Variants in Population-Scale Biobanks

**DOI:** 10.1101/2024.09.23.24314008

**Published:** 2024-10-07

**Authors:** David R. Blair, Neil Risch

**Affiliations:** 1. Division of Medical Genetics, Department of Pediatrics; 2. Department of Epidemiology & Biostatistics; 3. University of California San Francisco

## Abstract

Loss-of-function variants (LoFs) disrupt the activity of their impacted gene. They are often associated with clinical phenotypes, including autosomal dominant diseases driven by haploinsufficiency. Recent analyses using biobanks have suggested that LoF penetrance for some haploinsufficient disorders may be low, an observation that has important implications for population genomic screening. However, biobanks are also rife with missing data, and the reliability of these findings remains uncertain. Here, we examine the penetrance of putative LoFs (pLoFs) using a cohort of ≈24,000 carriers derived from two population-scale biobanks: the UK Biobank and the All of Us Research Program. We investigate several possible etiologies for reduced pLoF penetrance, including biobank recruitment biases, annotation artifacts, missed diagnoses, and incomplete clinical records. Systematically accounting for these factors increased penetrance, but widespread reduced penetrance remained. Therefore, we hypothesized that other factors must be driving this phenomenon. To test this, we trained machine learning models to identify pLoFs with high penetrance using the genomic features specific to each variant. These models were predictive of penetrance across a range of diseases and ploF types, including those with prior evidence for pathogenicity. This suggests that reduced ploF penetrance is in fact common, and care should be taken when counseling asymptomatic carriers.

## Introduction:

Exome and genome sequencing are now first-tier tests for rare disease diagnosis^1–6^. Given this success, there is growing interest in applying these technologies to asymptomatic patients^7–17^. The utility of sequencing for screening remains uncertain^18–20^. Generally, a screening test’s utility is quantified using its positive predictive value. For genetic testing, this statistic is driven both by the accuracy of the genotype call and its penetrance for the phenotype of interest, where penetrance is defined as the probability that a carrier will manifest the disease. Although imperfect, the accuracy of genotype calling is relatively high^21,22^. Alternatively, penetrance estimates for most genotypes are unknown; they can range anywhere from 0 (no associated disease risk) to 1 (certain disease manifestation). These estimates also vary with age and can be modified by additional factors, including polygenic background^23–25^ and environmental exposures^26^. For diagnostic applications, accurate penetrance estimates are less critical. Patients already express a disease phenotype, so laboratories typically must only determine if variants are pathogenic (i.e. disease-associated) or benign^27^. Variant interpretation for screening applications is more complex. Laboratories and clinicians should be able to express how likely the variants are to cause disease in the future. This risk is of course inextricably tied to penetrance.

Penetrance is notoriously difficult to estimate^28^. For a few pathogenic genotypes that are unusually common (typically due to founder events), accurate penetrance estimation is possible^29–32^. Generally, however, pathogenic genotypes are extremely rare. As a result, penetrance is unknown for most clinically relevant variants. Recently, population-scale biobanks with linked electronic health record (EHR) data have become widely available^33–40^, and these datasets have been used to estimate penetrance using a “genotype first” approach^41^. Here, pathogenic variant carriers are identified using the available genetic data, after which their phenotypic expression is assessed retrospectively using EHR data. These analyses have suggested widespread reduced penetrance for pathogenic variants^42–44^. This observation has important implications for genomic screening, as it suggests that the positive predictive value of genetic testing may be unacceptably low. That said, biobanks may have limitations as a resource for estimating penetrance, and biases related to recruitment, coupled with missing data, may lead to deflated estimates^41,45^.

Here, we investigate the apparent penetrance for one of the simplest classes of potentially pathogenic mutations: putative loss-of-function (pLoF) variants in haploinsufficient disease genes. To do so, we uniformly processed the genomic data from two biobanks (the UK Biobank^36^ and the All of Us Research Program^40^; combined N>700,000), identifying ≈24,000 pLoF carriers at risk for 91 diseases. We then analyzed their relative frequencies across biobanks and diseases, demonstrating that the types and frequencies of pLoFs in these biobanks were likely shaped by recruitment biases. Nevertheless, biobank pLoFs had strong and replicable phenotypic effects, and consistent with prior analyses, penetrance was generally reduced. We then investigated possible factors underlying the reduced penetrance, adjusting estimates accordingly. Examples include annotation artifacts, missed diagnoses, and censored clinical data. Accounting for all these factors increased estimates, but widespread reduced penetrance remained. Therefore, we hypothesized that many of these variants may in fact have intrinsically low penetrance, which may be a function of incomplete or “leaky” loss-of-function. To test this, we trained machine learning models to predict pLoF penetrance using variant-specific genomic features that may correlate with incomplete loss-of-function. These models were predictive of penetrance across a range of diseases and variant types, including those previously annotated to be pathogenic by diagnostic testing laboratories^46^. Consequently, LoF penetrance remains quite uncertain, and accurately communicating this uncertainty to asymptomatic carriers will be crucial for successful genomic screening.

## Results

### Biobanks are Likely Depleted of pLoFs with Severe Phenotypic Effects

**Figure 1:**
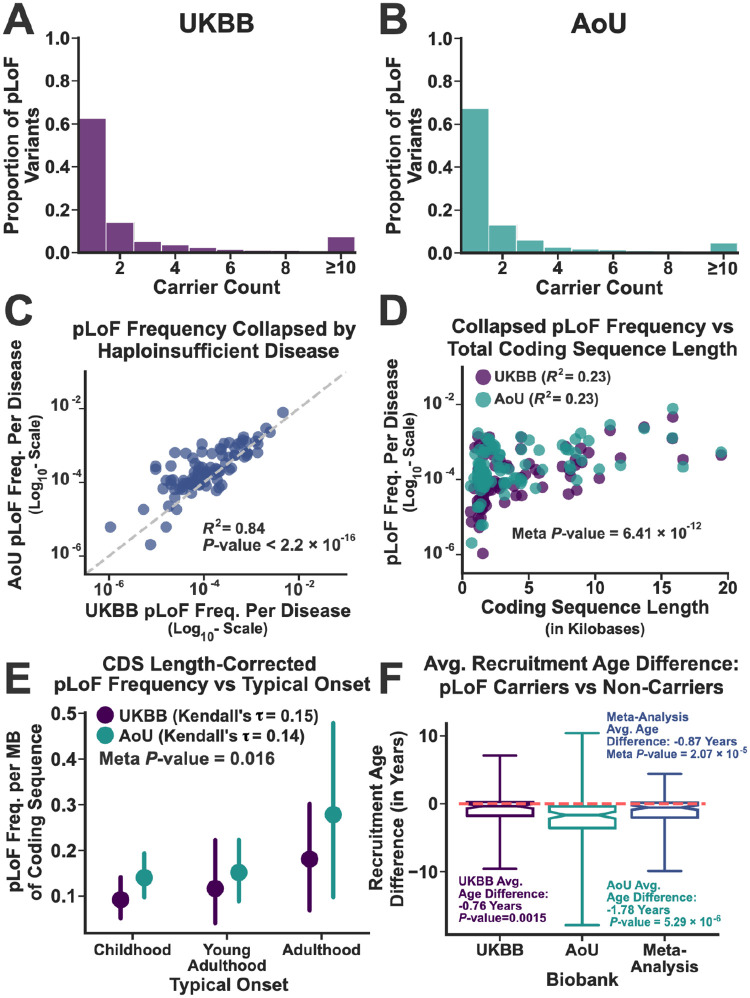
Biobank datasets are likely depleted of pLoFs with severe phenotypic effects. (A, B): The carrier count distributions for the pLoF variants identified in the UK Biobank (UKBB; B) and the All of Us Research Program (AoU; B). (C): The disease-specific pLoF carrier frequencies (N=91) compared across the two biobanks: UKBB (x-axis) and AoU (y-axis). Correlation was assessed using Pearson’s method $\left(R^{2}\right)$. (D): The coding sequence (CDS) length was computed using the MANE Select transcript for the gene(s) linked to each disease (x-axis), and this was compared to the per-disease pLoF frequency (y-axis) using Pearson’s method ($R^{2}$). (E): The collapsed pLoF frequencies were normalized by the CDS length (in megabases; MB) and plotted against the three onset classes. Correlation was assessed using Kendall’s rank correlation (denoted τ). Error bars represent 95% bootstrapped confidence intervals. (F): Boxplots display the distribution over the differences in recruitment age for the pLoF carriers and their corresponding non-carrier controls (see Methods). This distribution is depicted for each biobank along with a boxplot that summarizes the results of a cross-biobank meta-analysis. The edges of the box define the interquartile range, while the notch indicates the median. The whiskers depict the total range. Statistical significance was assessed using a Wilcoxon signed rank test (two-sided).

Using the ClinGen database^47^, we identified 91 autosomal dominant/pseudo-autosomal dominant Mendelian disorders for which haploinsufficiency is a likely mechanism of disease (see Methods). This set of diseases covered a broad range of human pathophysiology, including neurodevelopmental disorders, congenital malformation syndromes, and diseases linked to tumor predisposition. Most (76%) typically present during childhood while the remainder occur during various stages of adulthood. The specific diseases analyzed in this study, along with their annotated information, are provided in Supplemental Table 1. Following annotation, we linked the diseases to their associated genes using the Online Mendelian Inheritance in Man database^48^ (117 in total). We then systematically identified all putative loss-of-function variants (pLoFs) within these genes in both biobanks, removing those that likely represent technical artifacts based on sequencing depth and quality scores (see Methods). In total, we identified 3,131 unique pLoFs in the UK Biobank (UKBB; total N=468,672) and 3,889 in the All of Us Research Program (AoU; total N=245,376), resulting in a total of 6,247 unique pLoFs (773 occured in both). Additional details about the individual variants can be found in Supplemental Tables 2 (UKBB) and 3 (AoU). The distribution of pLoF carrier counts in both datasets are displayed in Figures 1A and B. Most variants were singletons (63% and 67% in the UKBB and AllofUs respectively), consistent with their likely negative impact on fitness and survival.

Even though most individual variants were singletons, the total number of pLoFs per disease was highly variable, ranging over nearly three orders of magnitude (Figure 1C). Moreover, the disease-specific pLoF frequencies were highly correlated across the biobanks ($R^{2}=0.84;$ *P*-value < 2.2×10^−16^). Many factors likely drive this shared variability, including properties specific to the populations that constitute each biobank and attributes of the diseases and variants themselves. For example, founder effects and genetic drift almost certainly contribute to the shared variability displayed in Figure 1C. However, the demographic backgrounds for the two biobanks are quite distinct^36,40^. The UKBB mostly contains subjects of European ancestry (≈90%)^36^, while AoU is far more diverse^40^ (European fraction ≈50%). Therefore, while founder variants likely drive some of the shared variability in per-disease pLoF frequencies, their contribution should be limited. Moreover, if genetic drift were driving the shared variability, the pLoF frequencies should correlate with the number of coding sites linked to each disease. While this was true (Figure 1D), the fraction of the variability explained by this phenomenon was only 23%, suggesting that other factors were likely involved.

The per-disease pLoF frequency estimates, after correcting for coding sequence length, were positively correlated with typical disease onset in both biobanks, such that pLoFs linked to childhood-onset diseases were less common than those linked to adult-onset disorders (Figure 1E). This suggested that the biobanks may be depleted of variants linked to childhood-onset diseases, likely due to recruitment biases that favor living and/or healthier adults. Notably, pLoF carriers in general were recruited at a younger age than their non-carrier counterparts (0.87 years on average, Wilcoxon Signed Rank Test Meta-Analysis *P*-value: 2.07×10^−5^; Figure 1F), consistent with a more pervasive recruitment bias that favors healthier individuals recruited prior to disease onset (see Supplemental Table 4 for all disease-specific results). This implies that biobanks that recruit younger subjects should harbor more pLoFs. The average recruitment age for AoU was 52 years compared to 57 years for the UKBB (*T*-test *P*-value <2.2×10^−16^), a consequence of the distinct recruiting strategies for two the studies^49,50^. Thus, it was not surprising to find that the overall pLoF carrier rates were higher in AoU than the UKBB (4.5% vs 3.0%; *P*-value <2.2×10^−16^, an effect that persisted after correcting for differences in ancestry (UKBB: 2.9%; AoU: 4.2%, see Methods).

To avoid such biases, penetrance estimates would ideally be derived from prospective cohorts with millions of subjects, either starting from birth or even during pregnancy. Given that such studies are currently infeasible, biobanks likely represent the best opportunity for systematic penetrance estimation. Based on the above analyses, however, these datasets are likely depleted of pLoFs with severe phenotypic effects, at least compared to younger cohorts. As corollary, they are likely enriched for variants with low penetrance and/or milder phenotypic effects. This implies that aggregate estimates of penetrance derived from biobanks may be systematically deflated when compared to those obtained using other study designs. This is likely to be particularly true for diseases associated with high morbidity and mortality. This does not necessarily imply penetrance estimates from these datasets are meaningless, but care should be taken when applying biobank penetrance estimates to other populations.

### pLoFs in Biobanks Have Detectable and Consistent Phenotypic Effects

**Figure 2:**
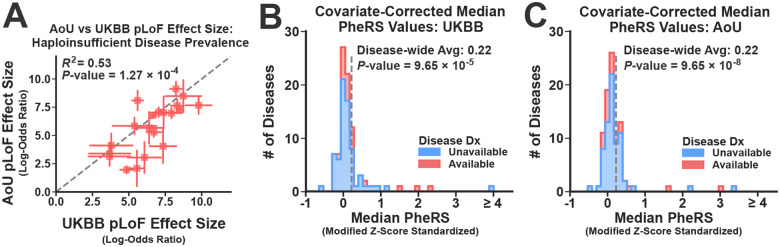
pLoFs have consistent average phenotypic effects in biobanks. (A): Average pLoF effects on disease prevalence are compared across the two biobanks (diseases with diagnoses available in both datasets; N=28). The datapoints represent the mean effect size (log-odds), while the bars indicate standard errors. Correlation was assessed using Pearson’s correlation coefficient ($R^{2}$). (B, C): The median Phenotype Risk Score (PheRS) was computed using the pLoF carriers for each disease and standardized to the score distributions observed among non-carriers. These histograms display the statistical enrichment of positive PheRS scores among pLoF carriers in the UKBB (B; N=90) and AoU (C; N=90). Statistical significance was assessed using a Wilcoxon signed rank test ($H_{0}$: median PheRS is symmetric about μ<0). Blue color: diagnoses unavailable. Red color: diagnoses available.

Although biobanks are likely depleted of pLoFs with severe phenotypic effects, many carriers in these datasets still manifest strong signs of disease expression. To illustrate, we constructed control groups for each disease by identifying biobank subjects that did not carry any rare variant (allele frequency ≤ 0.1%) in their associated genes (see Methods). We then systematically estimated the effects of the pLoF variants on haploinsufficient disease risk by comparing the disease prevalence among carriers and non-carriers using logistic regression. This analysis was limited to those diseases with at least 1 diagnosis in either the carriers or non-carriers across both biobanks (N=28). In a cross-biobank meta-analysis, the pLoF variants had a Bonferroni-corrected statistically significant effect on risk for over two-thirds (20/28) of the haploinsufficient diseases (see Supplemental Table 5 for the full set of results). Moreover, the risk estimates for the statistically significant associations were correlated across biobanks ($R^{2}=0.53;$ *p*-value =1.27×10^−4^, see Figure 2A).

These results indicate that pLoFs have strong and replicable effects on disease prevalence, but the analysis was limited to diseases with diagnostic codes in available in the EHR data. Most haploinsufficient diseases were not amenable to this analysis (N=63), as they lacked the diagnostic data needed to assess their risk. To overcome this limitation, we also quantified the disease expression of the ploF variants using covariate-corrected Phenotype Risk Scores (PheRS)^51,52^. These scores measure the extent to which a subject is a phenotypic outlier based on their pattern of expressed disease-specific symptoms, which is possible even in the absence of a formal diagnosis (see Methods). Figures 2B (UKBB) and 2C (AoU) display the distributions over the median PheRS estimates for each disease, which were standardized using the PheRS distributions observed in non-carriers (Methods). Although the average relative increase in scores among the pLoF carriers was modest (Average Standardized PheRS = 0.22 in both biobanks), the PheRS estimates were systematically increased among carriers across the full set of diseases (60/90 in the UKBB, 68/90 in AoU; Wilcoxon Signed-Rank Meta-Analysis *P*-value = 2.88×10^−11^). Moreover, many individual diseases achieved dataset-wide (14/89) or at least marginal (37/89) significance in a cross-biobank meta-analysis (see Supplemental Table 6 for full set of results). Therefore, the pLoF variants were associated with increased disease expression risk in at least a fraction of subjects for most diseases.

### Reduced pLoF Penetrance is Not Fully Explained by Annotation Artifacts

**Figure 3:**
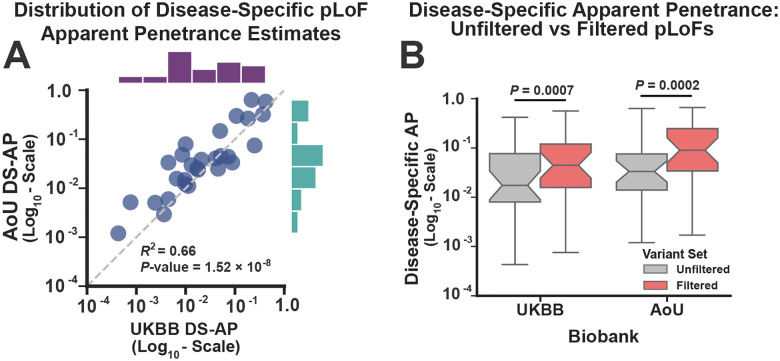
Reduced pLoF Penetrance is Not Fully Explained by Annotation Artifacts. (*A*): Disease-specific apparent penetrance (DS-AP) estimates for pLoFs (DS-APs, see Methods) were computed in each biobank using diagnoses only (N=28). The estimates from the two biobanks were compared, and their correlation was assessed using Pearson’s correlation coefficient (R^2^). (E): Boxplots comparing the distribution of DA-AP estimates before (gray) and after (red) filtering pLoFs for possible artifacts (see main text). Statistical significance was assessed using Wilcoxon signed rank tests (one-sided test; H_0_: difference in DS-AP after and before filtering is symmetric about μ<0).

Despite their evident effects on disease expression, the apparent penetrance of the pLoFs was not necessarily high. To estimate the average pLoF penetrance for each haploinsufficient disease, we measured their phenotypic expression using disease-specific diagnostic codes, generating point estimates and 95% confidence intervals using a simple binomial model (see Methods). Figure 3A compares the disease-specific average pLoF penetrance (DS-AP) estimates across the two biobanks, again focusing on those diseases with diagnostic data available in both biobanks (N=28). Clearly, this is an imperfect estimate of penetrance, as it makes strong assumptions about the sensitivity of diagnoses for measuring disease expression. Nevertheless, the DS-AP estimates were correlated across biobanks ($R^{2}=0.66;\;P$-value = 1.52×10^−8^), and consistent with previous analyses^43^, the median DS-AP estimates across diseases were reduced (1.7% ± Median Absolute Deviation=1.6% in UKBB; 3.3% ± 2.2% in AoU). Most studies that analyze pLoFs filter these variants to remove those that likely represent annotation artifacts^53–56^, which are variants that have no molecular impact but were misannotated as LoFs by the variant prediction software. To account for these artifacts, we repeated this analysis after removing variants that impacted non-canonical transcripts (i.e. non-MANE Select^57^) and/or failed to meet a set of quality filters (assessed using the LOFTEE package^54^). Restricting the analysis to the filtered variants increased the DS-AP estimates in both biobanks (23/28 diseases in UKBB; 24/28 in AoU; Wilcoxon Signed Rank Test Meta Analysis *P*-value = 9.54×10^−7^; see Figure 2E). However, their median values remained below 10% (4.6% ± 3.7% in UKBB; 9.0% ± 7.2% in AoU). Nevertheless, several diseases achieved apparent penetrance estimates exceeding 20% penetrance (ex: Hereditary Hemorrhagic Telangiectasia and Neurofibromatosis Type 1; see Supplemental Table 7 for details. Note: many penetrance estimates in AoU must be suppressed due to restrictions on data sharing).

### Reduced Penetrance Persists after Accounting for Missing Disease Diagnoses

**Figure 4:**
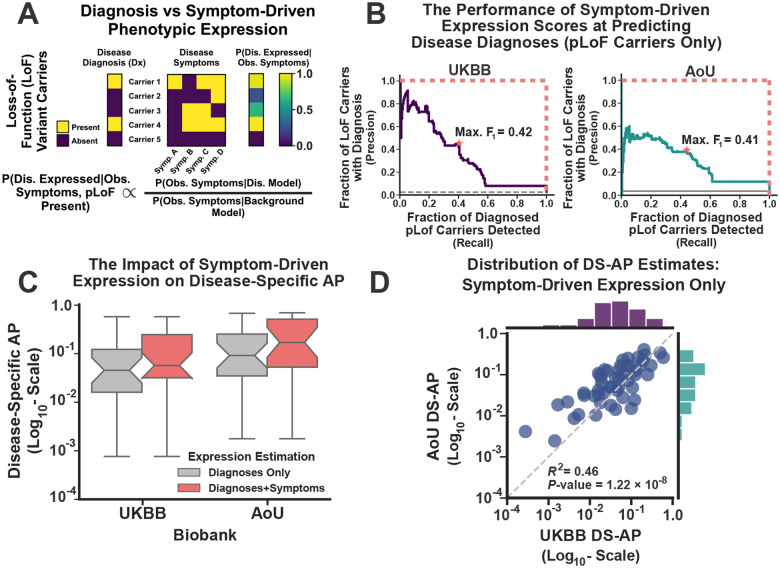
Reduced Penetrance Persists after Accounting for Missing Disease Diagnoses. (A): A simple illustration of the symptom-driven expression model. (B): Symptom-driven expression scores predict rare disease diagnoses in both datasets. These panels display the precision (i.e. fraction of LoF carriers with diagnoses; y-axis) and the recall (i.e. total fraction of pLoF carriers detected; x-axis) for a model that uses symptom-driven expression scores to identify pLoF carriers with disease diagnoses. Each point on these curves represents a different expression score threshold for identifying carriers at-risk for diagnosis. The red stars denote the expression score (≈0.97 in both datasets) that maximized the F_1_-measure (harmonic mean of (precision/recall) for the predictions. Gray lines indicate the performance of a random model; red-dashed lines the performance of a perfect classifier. The left panel depicts performance in the UKBB (training dataset; N=6,129 disease-carrier pairs), while the right panel depicts performance in AoU (validation dataset; N=8,242 disease-carrier pairs). Additional details are in the text. (C): For those diseases with diagnoses available in both biobanks (N=28), the two boxplots compare the disease-specific penetrance estimates before (gray) and after (red) including the symptom-driven scores (see Methods). (D): For those diseases without diagnostic data (N=51), the symptom-driven disease-specific APs for the pLoF variants were compared across the two biobanks. Correlation was assessed using Pearson’s method ($R^{2}$).

Thus far, diagnoses have been used to measure disease expression and estimate penetrance. This is clearly an imperfect method, as some pLoF carriers may exhibit disease expression without diagnoses^45,58^. Moreover, most haploinsufficient diseases lack disease-specific diagnostic codes that can be detected in EHR data. To overcome these issues, we developed an automated method to measure disease expression in every pLoF carrier using their recorded symptoms (see Figure 4A for illustration). Like the PheRS approach, this method computes the background symptom frequency distribution using the entire biobank (denominator in the right-hand-side of the equation in Figure 4A). To compute the probability of disease expression (conditional on being a pLoF carrier, left-hand-side of Figure 4A), the method compares the likelihood of the observed symptoms under a simple disease model (numerator in the right-hand-side) to this background distribution. This enables the method to compute a symptom-driven disease expression score for every pLoF carrier that accounts for their similarity to other affected carriers while comparing their expressed symptoms to a null background. This symptom-driven approach to measuring disease expression requires no labels and is thus unsupervised. However, it remains at risk for overfitting. Therefore, we trained the disease-specific expression models in the UKBB prior to validating them in AoU.

Evaluating the performance of the method is challenging, as no “gold-standard” disease expression dataset exists. Therefore, we used the haploinsufficient diseases with diagnostic data to validate the approach. Briefly, for those diseases with both symptom expression and diagnostic data, we used the symptom expression scores computed for each carrier to predict diagnoses, as they should be mostly concordant. Figure 4B depicts the results of this analysis using precision-recall curves. In these curves, each point on the line represents a distinct symptom-expression score. For each point, we identified those pLoF carriers with a symptom expression score that was at least as high. We then computed the fraction of LoF carriers within this set who harbored disease diagnoses (precision, y-axis) and compared this estimate to the total fraction of diagnosed LoF carriers detected within the subset (recall, x-axis). A perfect model would recover 100% of the diagnosed carriers with perfect precision (red-dotted lines). A random model could perform no better than the baseline disease diagnosis rate (gray dotted lines).

Clearly, the symptom-driven expression measurements predicted disease diagnoses significantly better than random (13-fold and 7.5-fold better in the UKBB and AoU, respectively; Randomization Test *P*-values < 0.0001, see Figure 4B). Moreover, there was not a substantial difference in model performance between the UKBB and AoU, although performance was certainly better in the UKBB (likely due to training bias). This suggests that symptom-driven expression scores are predictive of disease expression, at least according to diagnoses. To simply downstream analyses, we binarized the symptom-driven expression scores by selecting a single threshold for disease expression in each dataset. Those carriers with symptom scores above this threshold were deemed to have disease expression, while those with scores below the threshold were unexpressed. To determine this disease expression threshold, we chose the symptom scores that maximized the F_1_-measure for the curves shown in Figure 4B, where the F_1_-measure represents the harmonic mean of the precision and recall scores. Selecting a disease expression threshold in this manner is arbitrary without “gold-standard” data. But by using the symptom-expression score that maximized the F_1_-measure, we found no strong evidence for discordance between the symptom-driven measurements and those derived using diagnoses (McNemar’s Test *P*-values = 0.16 and 0.12 in the UKBB and AoU respectively).

To incorporate the symptom-driven measurements into penetrance estimates, we identified a pLoF as expressed if the carrier had a either disease diagnosis or if their symptom-driven score exceeded the F_1_ thresholds from Figure 4B. For the set of 28 diseases analyzed in Figure 3, incorporating the symptom-driven expression measurements increased the median DS-AP estimates by 1.64-fold and 2.01-fold in the UKBB and AoU, respectively (Figure 4C). This is not surprising and is consistent with the hypothesis that many pLoF carriers were symptomatic but lacked formal disease diagnoses. In addition, this symptom-driven approach allowed us to analyze 51 haploinsufficient diseases that lacked diagnostic information yet had symptoms that were shared across the two biobanks. For these diseases, the purely symptom-driven DS-AP estimates were correlated across biobanks (Figure 3E; $R^{2}=0.46;\;P$-value = 1.22×10^−8^). Nevertheless, overall pLoF penetrance was reduced (median DS-AP: 4.8% ± 3.5% and 9.2% ± 7.5% in the UKBB and AoU respectively), even for diseases with that had both diagnostic codes and symptom-driven expression data (5.2% ± 4.5% and 10.9% ± 9.9% respectively). The symptom-driven DS-AP estimates are provided in Supplemental Table 8.

### Reduced pLoF Penetrance Persists After Accounting for Gaps in EHR Data Coverage

Another factor that could negatively impact penetrance estimation is the incomplete lifetime coverage of the EHR data in biobanks^41,45^. More specifically, these datasets capture only a fraction of their subjects’ lifespans, and the data coverage for individual participants can vary widely. Therefore, extensive missing clinical data, which can occur during any lifetime interval (e.g. childhood, late adulthood, etc.), can give the false appearance of reduced penetrance. Supplemental Figure 1 shows the distributions over the age at the time of recruitment (1A,1E), the age of the earliest clinical observation (1B, 1F), the total number of documented clinical encounters (1C,1G), and the total number of years elapsed between the first and last encounters (1D,1H) for the pLoF carriers in the UKBB and AoU, respectively. Based on these distributions, it seems likely that clinical data coverage for some pLoF carriers was too low to reliably determine their disease expression. To test this hypothesis, we first determined whether each pLoF carrier expressed *any* symptom consistent with their associated haploinsufficient disease, identifying those without symptoms as being *completely asymptomatic*. We then used the attributes from Supplemental Figure 1 as features in a set of regression models designed to predict asymptomatic carriers, independent of any variant or gene information. If the models were accurate, then prediction scores derived from these data coverage statistics could be used to remove pLoF carriers with too little clinical data to make meaningful contributions to penetrance.

**Figure 5:**
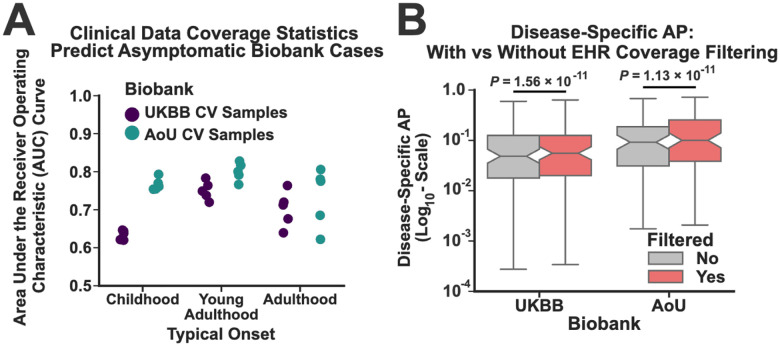
Correcting for clinical data coverage does not substantially increase penetrance estimates. (A): The coverage statistics displayed in Supplemental Figure 1 were used to build models to predict whether the pLoF carriers (stratified by disease onset; x-axis) would be asymptomatic for their target phenotypes. Model performance was assessed using the area under the receiver operating characteristic curve (AUC; y-axis) using leave-one-out 5-fold cross validation (CV). All models consistently performed better than random (AUC=0.5). (B): Based on the results from (A), pLoF carriers predicted to be asymptomatic at a false positive rate of 5% were removed from the analysis (see Methods). These boxplots depict the DS-AP estimates before (gray) and after (red) this filtering. Statistical significance was assessed using a Wilcoxon signed rank test (one-sided test; $H_{0}$: difference in DS-AP estimates after and before filtering are symmetric about μ<0).

To perform this analysis, we separated the diseases into three groups based on their approximate onset (see Methods): childhood, young adulthood, and adulthood, as we expect that these onset classes to have different coverage requirements (e.g. childhood-onset conditions may be poorly assessed in individuals without clinical data extending below age 20). We then fit and assessed the performance of these models in both biobanks using 5 -fold leave-one-out cross validation. The results are depicted in Figure 5A, where performance was assessed by comparing the model predictions to true asymptomatic status using receiver operating characteristic curves. For all three onset classes, the models were predictive of asymptomatic status, with some variability in performance across the different onset classes and biobanks.

Using the predictions from these models, we removed all subjects from our analysis who were predicted to be asymptomatic based on limited clinical data coverage at a false positive rate of 5% (see Methods). This process filtered out 17% and 35% of the pLoF carriers in the UKBB and AoU respectively. Figure 4B depicts the increase in DS-AP for the pLoFs within both datasets after correcting for clinical data coverage. DS-AP estimates increased for most diseases in both biobanks (65/90 in the UKBB; 64/76 in AoU; Wilcoxon Signed Rank Test Meta Analysis *P*-value < 2.2×10^−16^). Moreover, a handful of individual diseases acheived pLoF penetrance rates exceeding 50%, including Autosomal Dominant Polycystic Kidney Disease (Avg. Penetrance: 53% and 70% in the UKBB and AoU, respectively) However, this was not universally true, even for diseases that typically present during childhood (average pLoF penetrance for Tuberous Sclerosis: 6.6% and 19.2% in the UKBB and AoU respectively). Moreover, the absolute increase in DS-AP was modest, with the median penetrance estimates increasing 1.3-fold from those depicted in Figure 4 for both biobanks. The set of DS-AP estimates after filtering for clinical data coverage are provided as Supplemental Table 9.

### Variant-Specific Genomic Features Are Predictive of pLoF Penetrance

After removing annotation artifacts, imputing missed diagnoses, and correcting for incomplete clinical data coverage, pLoF penetrance estimates increased systematically. However, reduced penetrance remained common. None of the filters employed above were perfect, and residual artifacts, missed diagnoses, and incomplete data could certainly account for some fraction of the reduced penetrance. However, it’s certainly possible that some of the reduced penetrance was instead driven by pLoFs with incomplete loss-of-function. Generally, it is presumed that loss-of-function variants are approximately equivalent in terms of their molecular impact, but this may not be the case. Some may have deleterious effects but still result in some residual gene or protein activity. Such “leaky” expression could in turn drive variable penetrance. For example, incomplete non-sense mediated decay escape^59,60^ could allow for the partial expression of a transcript impacted by a stop-gain variant. Detecting such an effect for an individual variant is challenging, at least using biobank data. However, we hypothesize that variant-intrinsic genomic features (ex: SpliceAl scores for splicing variants^61^), which are often used to identify annotation artifacts, may also be predictive of penetrance. If true, then variant-intrinsic genomic features should be able to identify subsets of pLoFs with high penetrance, even among variants with prior evidence for pathogenic effects according to diagnostic testing data.

To test this hypothesis, we constructed machine learning models^62^ that used multiple variant-intrinsic genomic features to predict disease expression in individual pLoF carriers, where expression was measured using both disease-specific symptoms and diagnoses (see Methods for full description). Because the interpretation of different variant types relied on different features, we constructed unique models for each of the following pLoF classes: stop-gain, frameshift, and splice change (see Methods). These models were trained to predict disease expression exclusively within the UKBB (see Supplemental Figure 2 for a summary of the UKBB model training results), yielding variant expression prediction models that could be independently validated in AoU. To validate their effectiveness, we used the models to predict the expression risk for every pLoF variant in AoU. We then selected subsets of AoU pLoFs according to their predicted expression probabilities. If variant-intrinsic features were predictive of penetrance, then the average apparent penetrance of the pLoFs within the subsets should increase as the scores become more selective. At the same, increasingly restrictive expression scores will retain fewer expressed pLoFs, meaning that the total fraction of expressed pLoFs captured by the subset will decrease.

Figure 5A displays this tradeoff between average penetrance and retained fraction of expressed pLoFs over the range of machine learning (ML)-derived expression scores within the AoU validation dataset. For reference, we display this same tradeoff for the variants that pass the filter that we used in Figure 2 to remove annotations artifacts (MANE Select transcipts only^57^ with a high confidence LOFTEE flag^54^). A filter that only includes variants with non-conflicting pathogenic/likely-pathogenic (P/LP) annotations in ClinVar^46^ is also displayed. Clearly, the machine learning predictions can select ploFs with progressively increasing penetrance, and on average, the model prediction scores perform better than both the simple filter (ML Model Penetrance/Recall F_1_ measure = 0.26; MANE+LOFTEE F_1_ measure = 0.17; Bootstrapped *P*-value < 1.0×10^−4^) and prior P/LP annotations (ML Model Max. F_1_ measure = 0.26; P/LP F_1_ measure = 0.19; Bootstrapped *P*-value < 1.0×10^−4^). In Figure 5B, the results are stratified by variant type. The models were significantly predictive for all types (Randomization Test *P*-values < 1.0×10^−4^) but splice prediction was the most challenging (ML Model Avg. Penetrance Increase = 0.07). Figure 5C displays this same information, except now the results are stratified by disease onset. Even pLoFs in adult-onset haploinsufficient disease genes can be filtered to maximize penetrance (ML Model Avg. Penetrance Increase from Baseline = 0.09; Randomization Test *P*-value < 1.0×10^−4^).

Figure 5D displays this penetrance-recall tradeoff exclusively for variants classified as P/LP in ClinVar. The machine learning models remained strongly predictive of penetrance for this class of variants (ML Model Avg. Penetrance Increase from Baseline = 0.23; Randomization Test *P*-value < 1.0×10^−4^), with the most stringently filtered pLoFs approaching near complete penetrance. Based on this data, it’s unlikely that missing clinical data is accounting for much of the apparent variability in penetrance, as it’s hard to imagine why differences in clinical data coverage would correlate with variant-specific genomic features independently of their effects on disease expression. Instead, there are two much more likely (and non-mutually exclusive) explanations for this result^41^. First, many P/LP variants may have variable penetrance, which correlates with their genomic features. Second, the annotations in ClinVar may be incorrect. Distinguishing between these two possibilities is difficult, as most of these variants occur in only 1–2 subjects. Nevertheless, these results suggest that binary pathogenicity labels have low utility when it comes to predicting penetrance.

## Discussion

Broad (exome and genome) sequencing has revolutionized the field of clinical genetics^63^. More rare disease patients are being diagnosed using these technologies, and this has improved our ability to provide timely counseling and treatment to the affected individuals and their families. As a result, there is growing interest in using broad genomic testing for population screening^7–17^. Here, the goal is to identify individuals at risk for a Mendelian disease prior to symptom onset. Theoretically, this could lead to better clinical outcomes through earlier diagnosis, surveillance and management. For genomic screening to succeed, it should at least have a quantifiable positive predictive value^18–20^, a statistic that directly depends on penetrance. Currently, penetrance is almost universally unknown except for a handful of unusually frequent, deleterious variants. As a result, Mendelian disease risk assessments will be imprecise for most asymptomatic carriers. This may have a limited impact on patient outcomes in many settings. However, the clinical decisions made using genomic screening will be life altering in some cases, and without penetrance information, such interventions may be unnecessarily applied to low-risk carriers.

**Figure 6:**
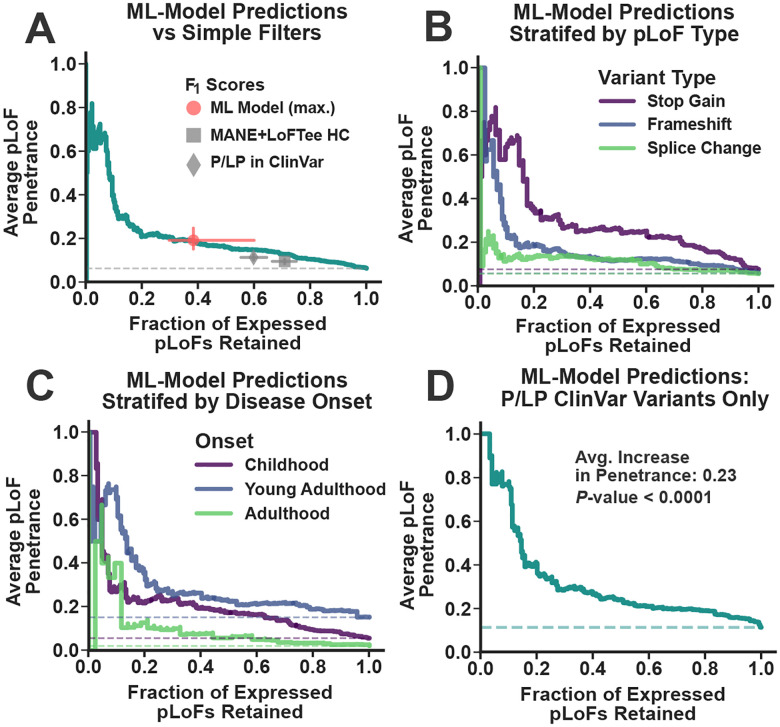
Genomic features are predictive of penetrance. (A): This panel displays the tradeoff between average apparent penetrance (y-axis) and the fraction of expressed pLoFs retained (x-axis) when using different model-derived expression prediction scores to filter variants. For reference, the threshold that maximized F_1_ measure for the model is shown in red. The performance of a simple filter (MANE Select+High-Confidence LOFTEE flag) is shown as a gray square, while a filter that only includes variants with nonconflicting pathogenic/likely-pathogenic (P/LP) annotations in ClinVar is shown as a gray diamond. All error bars represent bootstrapped 95% confidence intervals. The dotted line indicates the performance of a random classifier. (B, C): These panels depict the same penetrance- recall results from (A), except now pLoFs are stratified by variant type (B) or typical disease onset (C). (F): This panel depicts penetrance-recall curve for those pLoFs with non-conflicting P/LP annotations in ClinVar. Significance was assessed using a randomization test.

In this study, we investigated the penetrance of one of the simplest classes of clinically relevant genetic findings: putative loss-of-function variants (pLoFs) in haploinsufficient disease genes. Consistent with prior analyses^42,43,58^, we found that the apparent penetrance of these variants was reduced, with median values ranging from 5–10% depending on the biobank (Figure 3). Accounting for the extensive amount of missing clinical data in biobanks increased penetrance estimates (Figures 4 and 5), but most pLoFs remained unexpressed. In diagnostic applications, detailed criteria for variant interpretation have been developed to mitigate the risk for false positive results^27,64^. The utility of these criteria for penetrance prediction, however, is largely unknown. In this analysis, even variants with prior evidence for pathogenicity based on diagnostic testing had reduced penetrance (Figure 6A), suggesting that the utility of these annotations for penetrance prediction is limited. Importantly, machine learning models that incorporated variant-intrinsic genomic features like mutational constraint^65^, splicing scores^66^, and predicted non-sense mediated decay escape^59^ were able to identify pLoFs with penetrance approaching 100% (Figure 6D), indicating that missing clinical data alone was unlikely to account for the reduced penetrance for many these variants.

These results suggest that screening tests for these disorders that rely on current variant interpretation guidelines will have low positive predictive values. That said, improvements seem feasible. Decades of research into variant-intrinsic features like mutation patterns, evolutionary constraint, and functional impact has led to the development of computational tools that are used to predict the pathogenicity of individual pLoFs^54,55,59,65,66^, generally with the goal of eliminating annotation artifacts. However, the analyses performed in this study suggest that these tools may be effective at predicting variant penetrance, even in the absence of gene, disease, and carrier-specific information. In addition, their effectiveness at this task suggest that these tools may be capturing some degree of “leaky” or incomplete loss-of-function, which has implications for rare variant analyses beyond penetrance prediction. That said, the real-world clinical validity of these predictions remains unknown, and much work remains to be done to ensure that penetrance predictions derived from biobanks and similar resources are replicable, calibrated, minimally biased, and broadly applicable to diverse genes, diseases, and populations.

For now, it may be possible to predict the phenotypic outcomes for some rare genotypes with near complete penetrance^67^. In addition, when variant information is combined with orthogonal data like enzymatic activity and biomarkers, the prognostic accuracy may be very high^68^. Unfortunately, such assays are only available for a tiny fraction of genetic diseases. For most, the variants themselves are the only piece of prognostic information available. Prior evidence for pathogenicity may increase the positive predictive value of a particular variant, but based on the analyses presented here, prior pathogenic annotation labels are not synonymous with high penetrance, which is not unexpected. Access to high-quality outcome data for individual genotypes will certainly help, but given their intrinsically low frequency, it will likely remain difficult to estimate penetrance and predict phenotype outcomes in individual patients for the foreseeable future. Therefore, we suggest that caution be used when returning positive genomic findings to asymptomatic patients. Even with prior evidence of pathogenicity, risk estimates remain uncertain.

## Methods

### Haploinsufficient Disease Curation and Annotation

We used the ClinGen^47^ Database (downloaded on July 25th, 2023) to identify Mendelian disorders that have strong evidence to support haploinsufficiency as a mechanism of disease (ClinGen Dosage Haploinsufficiency Assertion Evidence Level 3). We then aligned these diseases to the Online Mendelian Inheritance in Man^48^ database (downloaded on February 23, 2023) using simple string matching followed by manual curation. This yielded 91 autosomal dominant/pseudo-autosomal dominant diseases linked to 117 genes, which were manually annotated with their typical onset (Childhood, Young Adulthood, Adulthood) and general classification (Congenital Malformation, Isolated Neurodevelopmental, Complex Neurodevelopmental, Tumor Predisposition, and Other) by a board-certified clinical geneticist (author D. Blair) using clinical expertise and literature review. Afterwards, disease-specific diagnostic codes were annotated to these diseases by manually curating the terminologies^69^ used by the Observational Medical Outcomes Partnership Common Data Model^70^ (OMOP-CDM). Finally, the diseases were annotated with a set of Human Phenotype Ontology^71^ (HPO) symptoms using the data from several ontologies, including the HPO itself (downloaded on February 21, 2023), the Disease Ontology^72^ (downloaded on February 23, 2023) and OrphaNet^73^ (downloaded on February 23, 2023 using the HOOM^74^ module). The sequence and transcript information for each of the 117 genes was downloaded from the Ensembl^75^ database (Release 109; GRCh38 assembly) using the PyEnsembl^76^ package. Additional gene and transcript information (exon-intron boundaries, 5’ and 3’ UTRs, full coding and amino acid sequences) was downloaded using gget^77^. The 91 haploinsufficient diseases, along with their annotated information, are provided in Supplemental Table 1.

### Aligning HPO Symptoms to the OMOP-CDM Terminology

To identify HPO^71^ symptom diagnoses in the EHR data, we needed to align this ontology to the structured diagnostic data available in the electronic health records of each biobank. Because both biobanks encode their clinical data using the OMOP-CDM^70^, we focused on aligning the HPO symptom terminology to the structured vocabulary used by this data model^69^. Unfortunately, aligning the HPO to other medical terminologies is largely an unsolved problem that lacks a consensus regarding best practices^78^. Therefore, we created a custom alignment by building on our previous work^24^ while implementing some new techniques.

First, we created an alignment map between the HPO and SNOMED-CT^79^, as the latter represents the most comprehensive medical terminology available for the dissemination of EHR data. It is also fully incorporated into the concept terminology used by the OMOP-CDM. To create an HPO-to-SNOMED map, we followed the approach of McArthur et al.^80^, who created a similar map between the HPO and PheCodes^81^. First, we constructed a map linking HPO to SNOMED-CT terms if they shared a common concept in the UMLS Metathesaurus^82^. Second, we used an ontology alignment algorithm (SORTA^83^) to find all SNOMED-CT terms that mapped to an HPO term with a similarity score of ≥ 0.8 for at least 1 of their associated string pairs (both SNOMED-CT and HPO often provide multiple strings for each term). For terms with multiple aligned string pairs, we collated all the similarity scores across the different string pairs, storing both an average and maximum score.

With an HPO-to-SNOMED map in place, the HPO terms themselves could be aligned directly to the concept terminology used by the OMOP-CDM, as a map from SNOMED-CT terms to the OMOP-CDM concepts is provided by Observational Health Data Sciences and Informatics (OHDSI) Collaborative^69,84^. However, it is important to note that one HPO term often mapped to multiple SNOMED-CT terms, which could then map to the OMOP terminology in multiple ways. Therefore, each HPO-OMOP alignment was often supported by multiple intermediary relationships. To summarize this phenomenon, we stored several pieces of information for each alignment that captured the quality of its supporting evidence. These included: the total number of intermediate relationships supporting the mapping, the fraction of these relationships that were supported by the UMLS, the fraction that achieved a SORTA string alignment similarity score ≥ 0.8, the average SORTA score across intermediaries, and maximum score achieved. In total, this process generated 35,825 unique HPO-to-OMOP alignments.

Because automated alignments like this tend to be rife with spurious results, one of the authors (D. Blair) manually reviewed 500 random mappings and annotated their medical accuracy. The accuracy was unsurprisingly variable, but overall, far better than random (average precision: 0.76). To further improve accuracy, we built a simple logistic regression classifier (implemented in sklearn^85^) to predict if an HPO-OMOP alignment was accurate. The model incorporated the alignment features described above as linear predictors (noting that the maximum achieved SORTA score was incorporated as interaction term with the total number of intermediate relationships). The model was trained on the 500 manually annotated alignments prior to being applied to the full dataset. In leave-one-out 5-fold cross validation experiments, the area under the receiver operator characteristic curve for the model predictions was 0.76 (standard error: 0.017), indicating that these predictions could provide a substantial improvement to alignment accuracy. Therefore, all ≈35,000 HPO-to-OMOP alignments were scored using the prediction model, and several false positive rate (FPR) thresholds were selected for downstream filtering. The complete set of HPO-to-OMOP Concept ID alignments (along with their features, manual annotations, machine learning scores, and whether they survived various FPR filtering thresholds) are provided as Supplemental Table 10. Finally, we experimented with various alignment FPR thresholds in downstream analyses. Overall, PheRS enrichment among pLoF carriers was highest when using the relationships that survived a 20% FPR threshold (data not shown). Therefore, this set of alignments was used for all the results reported in this manuscript.

### Sequence Data Quality Control, Variant Annotation, and Non-Carrier Cohort Identification

This study utilized the exome sequence (ES) data from the UK Biobank (UKBB)^36^ and the whole genome sequence (WGS) data from the All of Us (AoU) Research Program^40^ to investigate the penetrance of putative loss-of-function (pLoF) variants in haploinsufficient disease genes. For the AoU dataset, the WGS samples undergo an extensive quality control process, which ensures that samples meet several coverage and accuracy thresholds^40^. Therefore, all samples with WGS data that were not flagged by AoU’s quality control pipeline were analyzed in this study (N=245,376). For the UKBB, less sample-level quality control was performed a priori. Therefore, the ES data from this biobank underwent additional quality control filters consistent with those performed in previous studies^86^. Briefly, all samples that showed evidence for genetic and self-reported sex discordance (N=296), sample duplication (N=56), excessive SNP array-short read sequencing genotype discordance (N=513, including those that lacked array data), low read coverage for the haploinsufficient genes of interest (20x coverage at less than 90% of the base pairs; N=92), and excessive missing genotypes (N=329, again limited to the haploinsufficient genes of interest) were excluded from the analysis (total number of samples that failed quality control: 1,156). After excluding subjects that withdrew from the UKBB study, this dataset contained a total of 468,672 subjects with both ES and EHR data.

Following sample-specific quality control filtering, variants from the exonic regions of the haploinsufficient disease genes were isolated from both datasets (performed using bcftools^87^ in the UKBB and hail^88^ in AoU), storing the variant genotyping calls in VCF files. Individual-level data was then stripped from these files, and the predicted molecular effect of each variant was annotated using VEP^89^ (Version 110). Simultaneously, the variants were annotated with any previous interpretations documented within the ClinVar^46^ database (downloaded on May 13^th^, 2024). Finally, all) pLoFs within these datasets were identified using the LOFTEE^54^ plug-in for VEP, which also provided a flag indicating the overall confidence in this assessment (high vs low confidence; HC vs LC). Using the output from VEP, each pLoF was annotated with the its most clinically significant impacted transcript^57^ (MANE Select, MANE Plus Clinical, Other), and the variants were also assigned to one of three pLoF classes: stop-gain, frameshift, and splice change.

Following annotation, we returned to the VCF files that contained the individual-level genotype calls and isolated all pLoFs identified in the previous step. We then identified all carriers for each individual variant, removing those that did not meet a basic set of genotype-specific quality control filters^86^. For single nucleotide variants (SNVs), we assigned a no-call status to all carriers with a genotyping quality score < 30, sequencing depth < 7, and alternate allelic balance < 0.15. For insertion-deletion variants, we were more stringent and removed those calls with a quality score < 30, sequencing depth < 10, and alternate allelic balance < 0.2. In addition, we removed a variant from the analysis entirely if its call rate was <0.99 or if its carrier frequency was greater than 0.1% (after performing carrier-specific quality control). For the UKBB, we also a *priori* removed those variants that achieved an average read depth <10 for more than 10% of the samples in the dataset (per recommended best practices^90^). In total, this process identified 3,131 (Supplemental Table 2) and 3,889 (Supplemental Table 3) pLoFs carried by 14,010 and 11,022 subjects in the UKBB and AoU respectively. Note, some individuals harbored multiple pLoF variants within a single gene, suggesting the potential for *in cis* rescue versus sequencing artifacts. Such carriers were not excluded from basic pLoF frequency estimates (i.e. Figures 1) but were excluded from all other analyses. No further attempts were made to account *in cis* rescue events, and the extent of their impact on pLoF penetrance is a target for future work^56^.

Finally, for each haploinsufficient disease, we created a unique cohort of non-carrier controls that were unlikely to be at risk for the disease of interest. To do so, we first identified all subjects in both datasets that carried any rare variant (allele frequency ≤ 0.1%, performed using plink2^91^ or hail^88^) in the set of genes annotated to each disease. From the set of all possible control subjects, we then removed those that carried any rare variant in the target genes or had a no-call genotype at one of the pLoFs detected in those genes. The total number of non-carrier controls available for each disease was variable but exceeded 280,000 and 130,000 in all instances for the UKBB and AoU respectively.

### Recruitment Age Analysis

We hypothesized that the ascertainment biases intrinsic to biobank recruitment would result in differences in recruitment age between pLoF carriers and non-carriers. To test this, we first identified the recruitment age for every subject. For the UKBB, recruitment age is a specific entry in the dataset (Data-Field 21022). For AoU, we estimated recruitment age using the censored birthdate provided for each subject along with the date on which their genomic biospecimen was collected. The pLoF effects on recruitment age were estimated separately for each haploinsufficient disease using an ordinary least squares (OLS) regression model applied to the cohort of pLoF carriers plus their corresponding non-carrier controls:

(1) $${\vec{Y}}_{\text{Recruitment}\;\text{Age}}=\mu +\vec{G}\times \beta_{\text{pLoF}}+\epsilon ,$$

where $\vec{G}$ denotes a binary vector indicating pLoF carrier status, $\beta_{\text{pLoF}}$ indicates the disease specific pLoF effect on recruitment age, μ is an intercept term, and ϵ denotes a gaussian-distributed error term. Dataset-specific *P*-values were computed using a two-sided Student’s *T*-test (using the statsmodels^92^ package in Python), and inference results (${\overset{ˆ}{\beta }}_{\text{pLoF}},\;{\hat{\sigma }}_{\text{pLoF}}$) from the two biobanks were combined using a fixed-effects meta-analysis^93^. Disease-wide effects were quantified by taking mean of the pLoF effect estimates across diseases, and the significance of the disease-wide bias in recruitment age was assessed using a two-sided Wilcoxon Signed Rank Test. Results were combined across biobanks again using a fixed-effects meta-analysis. Supplemental Table 4 contains the complete set of results for the recruitment age analysis.

### Carrier Rate Analysis

Let $C_{i}=1$ indicate that the ith biobank subject is a carrier for at least one pLoF. Based on this definition, the pLoF carrier rate in each biobank is given by:

$$\text{Carrier}\;\text{Rate}=\frac{\sum_{i=1}^{N}c_{i}}{N}.$$

To correct carrier rates for genetic ancestry differences, we used the predicted ancestry labels provided by the All of Us Research program, which were assigned using a machine learning model trained on a set of reference samples with known ancestry^40^. We reproduced these ancestry assignments in UKBB using this same procedure. To correct carrier rates for ancestry differences, we included these ancestry labels as covariates in a logistic regression model to predict carrier status:

(1) $$\text{P}\left(\vec{C}|\rho ,\;\text{A},\;\vec{a}\right)=F\left(\rho +\text{A}\times \vec{a}\right),$$

where A denotes a matrix of predicted ancestry labels (for this project, predicted ancestry labels include African/African American, Admixed American, East Asian, South Asian, Middle Eastern, and European), $\vec{a}$ denotes the vector of their individual effects, and F(X) denotes the logistic function. The parameter ρ represents the baseline pLoF carrier rate after adjusting for ancestry.

### Haploinsufficient Disease Prevalence and Penetrance Analysis

The simplest way to measure pLoF phenotypic expression was using disease diagnoses. Let $\vec{D}$ denote a binary vector of length N, where in N is the number of pLoF carriers for some haploinsufficient disease of interest plus the number of non-carrier controls. Let $D_{i}=1$ denote that the ith subject was diagnosed with the disease of interest at least once in their EHR data, where diagnoses were identified using a set of manually annotated OMOP-CDM concept codes (see above). Finally, let $\vec{G}$ denote a binary vector indicating the pLoF carrier status for the N subjects. We estimated the biobank-specific pLoF effect sizes (log-odds ratios; denoted $\gamma_{\text{ploF}}$) using one of two approaches. For the more common diseases ($\sum_{i=1}^{N}D_{i}\geq 10$), we incorporated covariates into the analysis using the following log-linear model:

(2) $$\text{P}\left(\vec{D}|\mu ,\;\vec{G},\;\gamma_{\text{pLoF}},\;\text{X},\;\vec{a}\right)=F\left(\mu +\vec{G}\times \gamma_{\text{pLoF}}+\text{X}\times \vec{a}\right),$$

where F denotes the logistic function, μ is an intercept term, X is a matrix of covariates, and $\vec{a}$ is a vector of covariate effect size parameters. For the current study, we incorporated the following covariates into our analysis: recruitment age, birth sex, and the first 16 principal components of the genetic relatedness matrix. Model fitting was performed using Firth-penalized maximum-likelihood estimation^94^, and statistical inference was conducted using a likelihood ratio test. Even with Firth penalization, model inference returned spurious results when $\vec{D}$ was extremely sparse. Therefore, for very rare diseases ($\sum_{i=1}^{N}D_{i}<10$), we constructed 2 × 2 contingency tables from $\vec{D}$ and $\vec{G}$ and estimated the pLoF log-odds ratio and its corresponding standard error using the statsmodels^92^ software package in Python. *P*-values were estimated using Fisher’s exact test^95^. Finally, we performed cross-biobank meta-analyses of the pLoF effect sizes using the Cochran-Mantel-Haenszel Test for stratified contingency tables (again implemented in the statsmodels^92^ package). Supplemental Table 5 contains a summary of the results of our disease-specific prevalence association analysis.

To estimate the disease-specific pLoF apparent penetrance (DS-AP) estimates using diagnoses, we assumed a symmetric beta prior distribution over the DS-AP estimates with hyper-parameter θ=0.5. Assuming disease diagnoses among pLoF carriers follow a Bernoulli process, the posterior distribution over the DS-AP is:

$$\text{DS-AP}∼\text{Beta}\left(\theta +\sum_{i=1}^{N}D_{i}\times G_{i},\theta +\sum_{i=1}^{N}\left(1-D_{i}\right)\times G_{i}\right),$$

such that the average DS-AP estimate (denoted $\bar{\text{DS-AP}}$) is simply:

(3) $$\bar{\text{DS-AP}}=\frac{\theta +\sum_{i=1}^{N}D_{i}\times G_{i}}{2\theta +\sum_{i=1}^{N}G_{i}}.$$

In practice, $\bar{\text{DS-AP}}$ estimates were obtained in each biobank independently, allowing them to be compared across datasets (ex: Figure 3B).

### Phenotype Risk Score Analysis

Most haploinsufficient diseases lack structured diagnostic codes that can be used to identify their presence or absence in EHR data. In such instances, it can be difficult to determine if a subject is in fact expressing disease symptoms. Phenotype Risk Scores^51,52^ (PheRS’s) were developed to address this issue. These scores measure the extent to which a subject represents an outlier in phenotype space. Their effectiveness relies on a critical assumption: Mendelian disease patients should express constellations of symptoms that are highly atypical when compared to their unaffected counterparts. Although this is sometimes true, it is not the case for all diseases. Moreover, non-Mendelian disease patients can become phenotypic outliers as well, for example, if they develop unusual complications from a common disease or multiple common diseases at once. Therefore, PheRS’s are an imperfect method for assessing phenotypic expression, particularly if the goal is to separate Mendelian from non-Mendelian disease subjects based on symptom expression alone. Nevertheless, they are useful for determining if a pLoF carrier is potentially symptomatic.

Let ${\vec{S}}_{i}$ denote a binary vector of length K, where is K is the number of symptoms annotated to the Mendelian disease of interest. Let $S_{i,k}=1$ indicate that the kth symptom was diagnosed at least once in the ith subject’s EHR data. Finally, let $P\left({\vec{S}}_{i}|\theta \right)$ denote the probability of observing the set of symptoms diagnosed in the ith patient, where θ represents a set of parameters that define a background symptom expression probability model. The Phenotype Risk Score for the ith subject (denoted ${\text{PheRS}}_{i}$) is given by:

(4) $${\text{PheRS}}_{i}=-\text{log}\left[P\left({\vec{S}}_{i}|\theta \right)\right].$$

This formula is equivalent to the surprisal, or information content, of the diagnosed symptom set according to the model defined by θ, and it provides a measurement for how unusual or atypical this set of diagnosed symptoms is. For the approach to be effective, we must of course define the symptom expression probability model. Consistent with prior studies^51,52^, we assumed that the k th symptom occurs independently of the others according to a Bernoulli process defined by the parameter $\theta_{k}$. Therefore,

$$P\left(S_{i,k}|\theta_{k}\right)=\theta_{k}^{S_{i,k}}\times {\left(1-\theta_{k}\right)}^{1-S_{i,k}}.$$

To estimate the background model parameters, we assumed that the Mendelian disease cases were sufficiently rare in the general population such that their risk of biasing the symptom-specific parameter estimates (denoted ${\overset{ˆ}{\theta }}_{k}$) was negligibly low. Therefore, we estimated each symptom expression parameter independently using the maximum likelihood estimator for a Bernoulli process:

(5) $${\hat{\theta }}_{k}=\frac{\sum_{i=1}^{N}S_{i,k}}{N},$$

where N denotes the total number of subjects in the biobank. After estimating this expression model, the ${\text{PheRS}}_{i}$ score for each subject becomes:

(6) $${\text{PheRS}}_{i}=\sum_{k=1}^{K}-\text{log}\left({\overset{ˆ}{\theta }}_{k}\right)\times S_{i,k}-\text{log}\left(1-{\hat{\theta }}_{k}\right)\times \left(1-S_{i,k}\right).$$

In practice, we further adjusted the raw PheRS’s for confounding covariates (recruitment age, birth sex, and the first 16 components of the genetic relatedness matrix) using ordinary least squares regression.

After computing the covariate-adjusted disease-specific PheRS’s for every subject in both biobanks, we then compared the distribution of these scores between pLoF carriers and their non-carrier controls. To assign statistical significance, we used a one-sided Brunner-Munzel Non-Parametric Hypothesis Test (implemented in scipy^95^), which evaluated the null hypothesis that the PheRS’s observed in the pLoF carriers were stochastically less than those observed in controls. A fixed effects meta-analysis was performed using the effect size and standard error estimates produced by the Brunner-Munzel Tests performed in each biobank. Finally, for the histograms in Figures 2B and 2C, the median PheRS’s in the pLoF carriers were converted to modified Z-scores using the medians and median absolute deviations estimated within non-carrier controls. The complete set of PheRS results for all diseases are given in Supplemental Table 6.

### Estimating Symptom-Driven Disease Expression Scores

PheRS’s can suggest that a subject is a phenotypic outlier, but these scores do not necessarily provide an accurate assessment of whether a Mendelian disease is being expressed or not. For example, consider autosomal dominant polycystic kidney disease (ADPKD). Clearly, a pLoF carrier who has bilateral renal cysts complicated by chronic kidney disease is expressing the phenotype, but what if a carrier only experiences proteinuria? Proteinuria is certainly a symptom of ADPKD, so this carrier’s PheRS score will be greater than 0 . But proteinuria is an incredibly common symptom in the general population. Therefore, just because an ADPKD pLoF carrier experiences proteinuria at some point in their life doesn’t mean that they are expressing ADPKD.

To overcome this issue, we formulated the following symptom-driven disease expression model. As before, let $P\left({\vec{S}}_{i}|\theta \right)$ denote the probability that a set of symptoms ${\vec{S}}_{i}$ is being expressed according to some general background distribution. In addition, let $P\left({\vec{S}}_{i}|\delta \right)$ denote the probability that this symptom set is instead expressed within an individual affected by a Mendelian disease (where the parameter set δ defines the expression model). Finally, let $E_{i}=1$ indicate that the disease of interest is being expressed in the ith carrier. Consistent with the diagram in Figure 4A, the probability of disease expression in the ith pLoF carrier is given by:

(7) $$P\left(E_{i}=1|{\vec{S}}_{i},\;\delta ,\;\theta ,\;\pi \right)=F\left[\text{log}\left(\frac{\pi \times P\left({\vec{S}}_{i}|\delta \right)}{(1-\pi )\times P\left({\vec{S}}_{i}|\theta \right)}\right)\right],$$

where π is the prior probability of disease expression among all carriers and F is the logistic function.

For this symptom-driven expression model to be effective, both the disease-specific expression model (i.e. $P\left({\vec{S}}_{i}|\delta \right)$) and the expression prior probability (i.e. π) must be either known *a priori* or estimated from the data. Estimating π from the data is relatively straightforward, but the disease-specific expression model may be incredibly complex and is largely unknown. Moreover, the independence assumption invoked for PheRS estimation is unlikely to hold for Mendelian diseases, as it is the co-occurrence of multiple unusual symptoms that typically defines a Mendelian disease.

To overcome these issues, we assumed that $P\left({\vec{S}}_{i}|\delta \right)$ follows a completely arbitrary distribution over symptoms sets. More specifically, let δ define a multinomial distribution over all possible expressed symptom sets (i.e. all possible sets except the empty set), such that the distribution is defined by a parameter set with cardinality $2^{K}-1$, where K denotes the total number of symptoms annotated to some disease of interest. Clearly, even for modest values of K, the dimensionality of the model becomes unwieldly, so we made the simplifying assumption that the possible set of symptoms is much smaller than $2^{K}-1$ (i.e. many of the multinomial distribution parameters are equal to 0). Practically, we assumed that only $M_{\text{Obs}}+1$ symptom sets were possible, where $M_{\text{Obs}}$ denotes the number of unique symptoms sets observed across all biobank participants. The +1 term allows for the addition of a generic symptom set that accounts for any non-empty set that was not observed in the biobank, which enables the model to be trained in one biobank yet still be applicable to another. Note, the total complement of observed symptom sets in either biobank was very sparse compared to the cardinality of all possible sets, typically numbering in the 10s or 100s.

With this assumption in place, the Mendelian disease symptom expression model was defined as:

$$P\left({\vec{S}}_{i}=\vec{s}|\delta \right)=\sum_{m=1}^{M_{\text{Obs}}+1}\delta_{m}\times 1\left(\vec{s}\equiv {𝒮}_{m}\right)$$

where $1\left(\vec{s}\equiv {𝒮}_{m}\right)$ is an indicator function that returns 1 if and only if the observed symptom set $\vec{s}$ is identical to the symptom set whose expression probability is defined by $\delta_{m}$ (denoted ${𝒮}_{m}$ in the equation above). The symptom expression model defined in Eqn. 7 can then be used to specify the following likelihood for the observed symptom data:

(8) $$P\left(\text{S}|\text{E},\;\theta ,\;\delta ,\;\pi \right)=\prod_{i=1}^{V}\;\sum_{E_{i}\in 0,1}{\left[\pi \times P\left({\vec{S}}_{i}|\delta \right)\right]}^{E_{i}}\times {\left[\left(1-\pi \right)\times P\left({\vec{S}}_{i}|\theta \right)\right]}^{1-E_{i}},$$

where S denotes the matrix of diagnosed symptom sets across the V pLoF carriers. By estimating the model parameters (denoted $\overset{ˆ}{\theta },\;\overset{ˆ}{\delta }$, and $\overset{ˆ}{\pi }$) through likelihood maximization, the posterior probability over disease expression (defined in Eqn. 7) can be estimated.

For many diseases, the total number of pLoF carriers was small, making it difficult to simultaneously estimate both the disease-specific and background expression models simultaneously. Therefore, the background expression models used for PheRS estimation were used to define $P\left({\vec{S}}_{i}|\overset{ˆ}{\theta }\right)$ (see Eqn. 5 for estimation procedure). As a result, only the disease expression prior π and the parameters defining the disease-specific expression model (denoted δ) needed to be estimated. To regularize these estimates in the face of sparse data, we assumed that these parameters were drawn from uniform Beta and Dirichlet distributions respectively. The model specified in Eqn. 8 was then fit by maximizing a lower-bound on the marginal likelihood using variational Bayesian inference^96^. The posterior distributions over the individual expression probabilities, denoted $P\left(E_{i}=1|{\vec{S}}_{i},\;\overset{ˆ}{\delta },\;\overset{ˆ}{\theta },\;\overset{ˆ}{\pi }\right)$ for the ith carrier, were generated automatically during inference. In practice, we fit the disease-specific expression models (i.e. $\left({\vec{S}}_{i}|\delta \right)$) only within the UKBB (given its larger sample size), as such models remain at risk for overfitting even though inference is technically unsupervised. After fitting in the UKBB, the parameters estimated in this biobank were used to predict expression probabilities in AoU. Note, this procedure eliminated 15 diseases from our analysis, as these disorders did not share diagnosed symptoms across bioanks.

The previously described model generated expression probabilities for every eligible pLoF carrier. However, it did not ensure that these probabilities were calibrated to disease expression risk. In other words, if $P\left(E_{i}=1|{\vec{S}}_{i},\;\overset{ˆ}{\delta },\;\overset{ˆ}{\theta },\;\overset{ˆ}{\pi }\right)=0.5$, it was hard to determine what this meant from a disease expression perspective. To place these probabilities on a coherent scale, we turned to the set of diseases that have both diagnostic and symptom data available in both biobanks. If the symptom-driven model produced coherent expression probabilities, then these scores should be predictive of which pLoF carriers harbor Mendelian disease diagnoses.

To test this hypothesis, we used the symptom-driven expression probabilities to predict Mendelian disease diagnoses among pLoF carriers, computing the precision and recall scores across all possible symptom-driven expression probability thresholds. The results of this analysis are displayed in Figure 4B. Clearly, the symptom-driven expression probabilities performed better than random in both datasets. In addition, the performance of the expression probabilities was relatively consistent across the two biobanks, although performance was better in the UKBB. To a select a symptom expression threshold for downstream analyses, we identified the expression probability score that maximized the $F_{1}$ measure (harmonic mean of the precision and recall) for the predictions shown in Figure 4B. This threshold was nearly identical across the two biobanks (0.975 and 0.972 in the UKBB and AoU respectively). Importantly, after defining disease expression according to this threshold, the symptom-driven expression predictions were statistically indistinguishable from the disease diagnoses themselves (McNemar’s Test for paired nominal data, implemented in statsmodels^92^).

In all downstream analyses, we treated disease expression as a binary outcome. More specifically, we considered a pLoF to be expressed if the carrier harbored a Mendelian disease diagnosis (i.e. $D_{i}=1$, assuming diagnostic data was available) or if their symptom-driven expression probability (denoted $P\left(E_{i}=1|{\vec{S}}_{i},\;\overset{ˆ}{\delta },\;\overset{ˆ}{\theta },\;\overset{ˆ}{\pi }\right)$) exceeded the F_1_ thresholds described above. Treating disease expression as binary enabled us to estimate DS-AP’s using the same methods that were used for simple diagnoses (see Eqn. 3 for details). It also greatly simplified the machine learning analyses, as models for binary prediction are well-established. Additional work is needed to effectively incorporate the uncertainty that is inherent to measuring Mendelian disease expression into the types of analyses performed in this study.

### Strategy for Removing Samples with Incomplete Clinical Data Coverage

Biobanks are rife with incomplete clinical data, as the EHR is an imperfect representation of a patient’s phenotype. Moreover, biobank subjects are enrolled into these studies well into adulthood, and there is no guarantee that the records captured by the study represent their complete clinical history. Supplemental Figure 1 illustrates the extensive variability in data coverage that was observed within the UKBB and AoU. Given the limited data available for many of these subjects, phenotypic imputation was unrealistic. Therefore, we devised a method to flag and remove subjects from our analysis that had unacceptably low clinical data coverage.

Let $\vec{A}$ denote a binary vector of asymptomatic indicators, where $A_{i}=1$ indicates that the ith ploF carrier had no evidence for disease expression based on disease-specific diagnostic code(s) and/or documented symptoms (i.e. had no disease-relevant diagnoses). Moreover, let W denote a matrix of clinical data coverage statistics. For this analysis, we used the following four statistics to define data coverage: Age at First Clinical Encounter, Age at Recruitment, Total Number of Documented Clinical Encounters, and Age at Last Clinical Encounter. These four statistics provided a basic summary of a patient’s interaction with the healthcare system, at least according to the information in the biobanks. Finally, let $\vec{b}$ denote a vector of coverage statistic effect size parameters. We modeled the probability of phenotypic *non*-expression (i.e. asymptomatic status) conditional on clinical data coverage using the following log-linear model:

$$\text{P}(\vec{\text{A}}|\mu ,\;\text{W},\;\vec{b})=F(\mu +\text{W}\times \vec{b})$$

where F denotes the logistic function and μ is the intercept term. Because different diseases will have different coverage requirements (depending on their onset, pathophysiology, etc), we fit three versions of this model in both biobanks by grouping diseases together based on their typical onset.

More specifically, each version of the model was repeatedly fit in both biobanks using leave-one-out 5 -fold cross validation (model fitting was performed using the LogisticRegression function available in sklearn^85^ using the default hyperparameters). For each iteration, 80% of the onset-grouped pLoF carriers were used to estimate the parameters for the disease non-expression model. The remaining 20% were used for validation. Model performance was assessed using the area under the receiver operating characteristic curve. All models performed better than random, but there was considerable variability in their performance across typical onset and biobanks. To flag ploF carriers with insufficient clinical data, we identified the 5% false positive rate threshold in each validation subset. If a subject in a validation subset had a non-expression probability that exceeded this threshold, they were flagged for removal from downstream analyses. As discussed in the main text, this ad *hoc* procedure removed a substantial fraction of pLoF carriers from both datasets (17% and 35% in the UKBB and AoU respectively). Moreover, the average pLoF penetrance estimates increased systematically after filtering. Nevertheless, the absolute increase in phenotypic expression that occurred because of this filtering was low.

### Predicting pLoF Phenotypic Expression using Variant-Specific Features

Let ${\vec{V}}_{i}$ denote a vector of genomic features that characterize the pLoF variant carried by the ith subject. Examples of such features include its relative position within the amino acid sequence^97^ or its deleteriousness based on computational prediction tools^65,66^. The goal of this analysis is to predict the probability of phenotypic expression directly from the set of features that are unique to the pLoF carried by the ith subject:

$$P\left(E_{i}=1|{\vec{V}}_{i},\theta \right)=ℱ\left({\vec{V}}_{i};\theta \right)$$

where ℱ is some function that maps the vector ${\vec{V}}_{i}$ onto disease expression probability space via a parameter set θ. Practically, different models can accomplish this goal. For this study, we constructed ℱ using the random forest algorithm^62^ implemented in the sklearn^85^, which builds predictive models via an ensemble of individual decision trees. Model fitting was performed by minimizing the logarithmic loss function of the prediction model when applied to a cohort of training carriers (training algorithm hyperparameters: min_samples_leaf=5, min_samples_split=10, n_estimators=500). Note, we also considered simpler methods for constructing ℱ (i.e. penalized logistic regression) but found that they performed systematically worse than this ensemble learning approach (see Supplemental Figure 2), likely due to the latter’s ability to capture non-linear effects.

Any predictive model built using machine learning is at risk for overfitting, particularly models with many free parameters like random forests. To minimize the risk for overfitting, machine learning model inference was performed exclusively in the UKBB, after which the models were independently evaluated in AoU. In addition, only completely asymptomatic pLoF carriers were included as negative cases in the UKBB training dataset to avoid confounding the model with carriers who were weakly symptomatic but did not reach the severity threshold required to designate them as phenotypically expressed. Given that the two biobanks were recruited from the populations of two different countries, the risk that the validation dataset was contaminated with subjects from the training dataset was very low.

Finally, different types of pLoF variants have distinct features that likely impact their risk for expression^55^. Therefore, distinct phenotypic expression models were constructed for each of the three variant types analyzed in this study (stop gain, frameshift, and splice change). The remainder of this section describes the variant-specific features that were used to build phenotype expression prediction models for each class of pLoFs. These features rely heavily on the ideas from prior studies^55,97,98^.

#### Variant Class Agnostic Features:

- CADD Score^65^: The Combined Annotation-Dependent Depletion (CADD) score predicts the deleteriousness of individual variants using a single numerical score derived from a wide range of variant-specific features, including but not limited to evolutionary conservation, DNA sequence motifs, and predicted impact on biochemical activity. Uniquely, CADD does not build these scores by training on a set of variants known to cause human disease. Instead, the scores are inferred by fitting a machine learning model to a set of evolutionarily neutral variants (proxy-negative cases) and a set of simulated mutations, which may or may not be deleterious (proxy-positive cases). This makes CADD well-suited for the analysis conducted in this study, as the score should not be polluted with information from prior ClinVar annotations.
- LOFTEE Confidence Flag^54^: The LOFTEE plug-in for VEP^89^ not only identifies putative loss-of-function variants but also assigns them a confidence flag (low or high) based on several variant-specific features (e.g. distance from end of transcript, ancestral alleles, etc.; see https://github.com/konradjk/LOFTEE for details)
- Transcript Type^57^: All variants were assigned to one of three transcript types (MANE Select, MANE Plus Clinical, Other) based on the most clinically relevant transcript that was predicted to be impacted.

#### Stop-Gain Variant Features:

- Predicted Non-sense Mediate Decay (NMD) Escape^60^: It is well known that some stop-gain variants escape non-sense mediated decay, enabling the expression of a potentially functional but truncated transcript. To predict NMD escape, we used the decision tree developed in Lindeboom et al^59^. Note, we did not encode predicted NMD Escape using a binary annotation (Present, Absent) but instead included the reason for the predicted escape into the model (No NMD Escape Present, Last Exon, First Exon ≤ 150nt from Start, Large Exon, ≤50nt from Last Exon-Exon Junction).
- Predicted Fraction of Amino Acids Lost^97^: If a variant escapes NMD, this feature computes the fraction of the amino acid sequence predicted to be lost. For stop-gain variants, this is simply it’s relative distance from the N -terminus (according to the MANE Select^57^ transcript).
- Possible Methionine Rescue (Translation Re-initiation)^55^: If a stop-gain variant occurs early enough in the amino acid sequence, then translation can potentially be rescued by another methionine residue that occurs just downstream. The exact criteria needed to be met for this to occur are unknown and may be variable across proteins. For this analysis, a stop gain variant had to meet the following criteria to flag for possible methionine rescue: 1) located in the first exon and 2) have a downstream methionine for alternate translation initiation that truncated <10% of the total protein length.

#### Frameshift Variant Features

- Last Coding Exon^55^: This is a simple binary feature that indicates if the frameshift variant occurred in the last exon.
- Predicted Fraction of Amino Acids Impacted^97^: This feature computes the relative fraction of amino acids predicted to be lost by an expressed frameshift. Like stop-gain variants, this feature captures the relative distance from N -terminus of the protein for the last normal amino acid.
- Possible Methionine Rescue^55^: This feature is computed in the same fashion for frameshift and stop gain variants.
- Note, NMD escape is certainly possible for frameshift variants with the added complexity that the escape is occurring on a frameshifted sequence. It’s possible that additional features based on NMD escape would improve frameshift penetrance prediction, but additional work is needed to determine when these rules may apply.

#### Splice Change Variant Features

- SpliceAI Score^66^: SpliceAI is a deep learning model that predicts changes in the splicing probabilities at different sites induced by a genetic variant relative to the splicing probabilities for the reference sequence (assuming some specific transcript model). For the current analysis, we re-computed SpliceAl scores using the Ensembl transcripts for each gene (Release 109), allowing for a maximum 500bp between the variant and impacted site. For expression prediction, the maximum SpliceAl score (maximum difference in splicing probability between the reference and mutated transcript) was included as feature. Note, several additional features were derived for splice variants based on the SpliceAl output. These are outlined in detail below.
- Predicted Fraction of Amino Acids Lost^97^: Like the other variant classes, we computed the fraction of amino acids that would theoretically be lost based on the splice site location assuming that it was expressed rather than undergoing NMD. Determining the exact location of the last normal amino acid for splice variants can be challenging. Therefore, we set the last normal amino acid to be the residue just proximal to the impacted splice site in the transcript model. This could clearly be improved (ex: by considering in-frame splice rescue events, exon skipping, etc), but this will be the focus of future work.
- Splice Mutation Type: Pathogenic splice mutations can impact transcript structure in complex ways, sometimes inducing multiple changes simultaneously. For the sake of simplicity, we used the SpliceAl output to assign each mutation to one of five classes based on the highest SpliceAl score observed for the variant: Donor Gain, Donor Loss, Acceptor Gain, Acceptor Loss and Indeterminate (i.e. maximum SpliceAl score = 0.0 or NaN).
- Outside Coding Region: Some splice sites occur in exons that lie outside the coding region. Although they could result in loss-of-function, many of these may be tolerated. Therefore, we included a binary feature that flagged splice variants predicted to impact only non-coding exons.
- Last Coding Exon^55^: This feature indicates whether a splice mutation is predicted to impact the last coding exon. Like the other variant classes, such mutations should be more likely to be tolerated.
- Persistent Original Splice Site Score: Sometimes, SpliceAl predicts that the original splice site remains intact with some non-zero probability, which may be indicative of leaky wild type expression. Therefore, we computed the difference between the SpliceAI score for the original and derived sites. Generally, this is simply equivalent to the global SpliceAl score, but other times, a variant increases the splicing probability for the wildtype splice site along with the derived site. This feature accounts for this phenomenon.
- In-frame Exon Rescue^55^: If the exon impacted by a splice change has a nucleotide length that is a multiple of 3 , then it can theoretically be skipped without disrupting the reading frame. This phenomenon was accounted for in the model using a binary feature (Present, Absent).
- Possible Methionine Rescue^55^: For splice variants, this is a less likely rescue mechanism. Nevertheless, given that a variant impacted the first exon, we allowed for possible methionine rescue assuming that there was a methionine residue in the second exon that truncated less than 10% of the amino acid sequence.
- In-frame Intron Retention^55^: If the intron to be spliced out has a nucleotide length that is a multiple of 3, then it can potentially be retained without impacting the transcript reading frame. This phenomenon is accounted for in the model using a binary feature (Present, Absent).
- Cryptic Rescue Score^55,98^: Many times, when SpliceAl predicts a primary splice site change, a secondary change is predicted to occur simultaneously that could negate the impact of the primary. More specifically, if a genetic variant is predicted cause a donor (acceptor) loss event in a transcript, there can be a complementary donor (acceptor) gain event just upstream/downstream of the predicted loss site but with a lower SpliceAl score. If this event remains in-frame with the original transcript, then the impact of the mutation may be minimal, as this complementary site could compensate for the loss. Alternatively, many donor (acceptor) gain events occur in-frame with the original donor (acceptor) site. So as long the downstream acceptor (upstream donor) site remains intact, then the impact of the variant may be insignificant. This Cryptic Rescue Score summarizes both possible rescue events using the output from SpliceAl. For primary splice site loss events (donor or acceptor), the Cryptic Rescue Score is simply the SpliceAl score for the in-frame gain event (assigned 0.0 if no in-frame gain is predicted). For primary in-frame gain events (donor or acceptor), the Cryptic Splice Score is harder to define. For this analysis, we used the corresponding splice site loss score (ex: a loss score of 1.0 should indicate that this predicted in-frame gain is preferentially being used) but acknowledge that this very much imperfectly captures the phenomenon. Clearly, more work is needed to effectively capture the complexity of splice mutation rescue events.

### Additional Statistical Methods

Unless otherwise noted, the statistical analyses described in the main text and/or figure legends were performed using the implementations (sometimes with slight modification) available in the following Python packages: scipy^95^, sklearn^85^, statsmodels^92^, and pandas^99^. Bootstrapped hypothesis testing was performed by generating empirical distributions for the target parameter estimates using re-sampling with replacement (10,000 re-samples for all tests). Randomization tests were performed similarly. All meta-analyses were performed using a fixed-effects model based on the standard normal distribution^93^.

## Supplementary Material

Supplement 1

1

## Data Availability

The genomic and electronic health data used for this analysis are publicly available but have strict data use agreements. The process for obtaining access to these biobanks can be found on their respective websites: https://www.researchallofus.org/register/ (All of Us Research Program) and https://www.ukbiobank.ac.uk/enable-your-research/register (UK Biobank). Haploinsufficient disease annotations are provided in Supplemental Table 1. The custom HPO-to-OMOP concept alignments generated in this study are provided as Supplemental Table 10. All other databases used in this analysis are freely available in the public domain.
