# Reduced Penetrance is Common Among Predicted Loss-of-Function Variants and is Likely Driven by Residual Allelic Activity

**DOI:** 10.1101/2024.09.23.24314008

**Published:** 2025-03-20

**Authors:** David R. Blair, Neil Risch

## Abstract

Loss-of-function genetic variants (LoFs) often result in severe phenotypes, including autosomal dominant diseases driven by haploinsufficiency. Due to low carrier frequencies, their penetrance is generally unknown but typically variable. Here, we investigate the penetrance of >6,000 predicted LoFs (pLoFs) linked to 91 haploinsufficient diseases using a cohort of ≈24,000 carriers with linked electronic health record data. We find evidence for widespread reduced penetrance, which persisted after accounting for variant annotation artifacts, missed diagnoses, and incomplete clinical data. We thus hypothesized that many pLoFs have incomplete penetrance, which may be driven by residual allelic activity. To test this, we trained machine learning models to predict pLoF penetrance using variant-specific genomic features that may correlate with incomplete loss-of-function. The models were predictive of pLoF penetrance across a range of diseases and variant types, including those with prior clinical evidence for pathogenicity. This suggests that many pLoFs have incomplete penetrance due to residual allelic activity, complicating disease prognostication in asymptomatic carriers.

## Introduction:

Exome (ES) and genome sequencing (GS) are now first-tier tests for rare disease diagnosis[1–6]. Given this success, there is growing interest in applying these technologies to asymptomatic individuals[7–17], including those incidentally found to carry pathogenic variants during routine diagnostic testing[18]. The utility of sequencing for prognosis remains uncertain[19–21]. Generally, a test's utility for prognosis is quantified using its positive predictive value. For genetic testing, this statistic is driven by both the accuracy of the genotype calls and their penetrance, where penetrance is defined as the (age-dependent) probability that a carrier will manifest disease. Although imperfect, genotype calling accuracy is relatively high[22,23]. Alternatively, penetrance for most genotypes is unknown. It can range from 0 (no associated disease risk) to 1 (certain disease manifestation), vary with age, and be modified by additional factors, including polygenic background[24–26] and environmental exposures[27]. For diagnostic applications, accurate penetrance estimates are not critical. Patients already express a disease phenotype, so laboratories must only determine if variants are causal (i.e. pathogenic) or benign[28]. Variant interpretation in asymptomatic cases is more complex. Laboratories and clinicians should be able to express how likely the variants are to cause disease in the future. This risk is, of course, inextricably tied to penetrance.

NOTE: This preprint reports new research that has not been certified by peer review and should not be used to guide clinical practice.

Penetrance is notoriously difficult to estimate[29]. For a few pathogenic genotypes that are unusually common (typically due to founder events), accurate penetrance estimation is possible[30–33]. Generally, however, pathogenic genotypes are extremely rare. As a result, penetrance is unknown for most. It is tempting to generalize penetrance estimates from a single or few well-characterized variants to others within the same gene, particularly for those variants predicted to have similar molecular impacts (e.g. loss-of-function). However, there is little evidence to support this strong assumption. Recently, population-scale biobanks with linked electronic health record (EHR) and ES or GS data have become widely available[34–41]. These datasets have been used to estimate penetrance using a “genotype-first” approach[42]. Here, pathogenic genotype carriers are identified using the available genetic data, after which their disease expression is measured retrospectively using EHR data. Although imperfect, these analyses suggest widespread incomplete penetrance for pathogenic genotypes[43–45]. This observation has important implications for the interpretation of genetic testing in asymptomatic individuals, as it suggests that the positive predictive value for many genotypes will be unacceptably low. That said, biobanks also have limitations as a resource for estimating penetrance, as fragmented phenotypes and missing clinical data, along with variant annotation artifacts, may lead to deflated estimates[42,46].

Here, we investigate the range of apparent penetrance for one of the simplest classes of potentially pathogenic mutations: predicted loss-of-function (pLoF) variants in haploinsufficient disease genes. To do so, we uniformly processed the genomic data from two biobanks (the UK Biobank[37] and the All of Us Research Program[41]; combined $N$>700,000), identifying ≈24,000 pLoF carriers at risk for 91 diseases. On average, biobank pLoFs were associated with disease expression, but consistent with prior studies, penetrance was generally low. We investigated factors that may underly the apparently reduced penetrance, including annotation artifacts, missed diagnoses, and incomplete clinical data. Accounting for these factors increased penetrance, but widespread reduced penetrance persisted. Therefore, we hypothesized that many of these variants may have intrinsically reduced or no penetrance, driven by residual or “leaky” allelic activity. To test this, we trained machine learning models to predict pLoF penetrance using variant-specific genomic features that might correlate with incomplete loss-of-function. These models were able to stratify pLoFs according to their penetrance risk for a range of diseases and variant types, including those previously annotated as pathogenic by diagnostic testing laboratories. This suggests that variants classified as LoF may in fact have varying degrees of residual function[47], and consequently, their penetrance remains uncertain. Although these analyses were limited to pLoFs in haploinsufficient disease genes, our results likely generalize to other types of disorders (ex: autosomal recessive), variant types (ex: missense) and genetic mechanisms (ex: gain-of-function). Accurately communicating this uncertainty will be crucial for the success of prognostic genomic testing, including population-scale screening programs.

## Results

### *Haploinsufficient Disease and pLoF Identification*

Using the ClinGen database[48] (Figure 1A; Step 1), we identified 91 autosomal dominant/pseudo-autosomal dominant Mendelian disorders for which haploinsufficiency is an established mechanism of disease (see Methods). This set of diseases covered a broad range of human pathophysiology, including neurodevelopmental disorders, congenital malformation syndromes, and diseases linked to tumor predisposition. Most (76%) typically present during childhood while the remainder occur throughout adulthood. Figure 1B depicts a summary of the 91 diseases. The individual diseases, along with their annotated information, are provided as Supplementary Dataset 1.

Following annotation, we linked the diseases to their associated genes using the Online Mendelian Inheritance in Man database[49] (117 in total; Figure 1A, Step 2). We then systematically identified all rare (biobank-specific carrier frequencies $\leq$ 0.1%), predicted loss-of-function variants (limited to stop gains, frameshifts and splice changes; pLoFs) within these genes in both biobanks, removing those that likely represent technical artifacts based on sequencing depth and quality scores (Figure 1A, Step 3; see Methods for details). In total, we identified 3,131 unique pLoFs in the UK Biobank (UKBB; total $N$= 468,672) and 3,889 in the All of Us Research Program (AoU; total $N$= 245,376), resulting in a total of 6,247 unique variants (773 occurred in both biobanks). The individual variants, along with their annotations, are provided as Supplementary Dataset 2 (UKBB) and Supplementary Dataset 3 (AoU). The distributions of pLoF carrier counts for the two biobanks are displayed in Figures 1C and 1D. Most were singletons (63% and 67% in the UKBB and AoU, respectively). Nevertheless, the marginal pLoF carrier frequencies were surprisingly high: 3.0% and 4.5% of subjects in the UKBB and AoU carried at least one pLoF in one of the 117 genes. The substantial difference in carrier frequencies across the two biobanks likely reflects their distinct demographies[37,41] and ascertainment practices[50,51]. Regardless, these carrier frequencies were much too high for a set of variants that consistently cause disease, suggesting that many had little-to-no phenotypic impact, particularly since they represent only a tiny subset of all possible disease-associated variants.

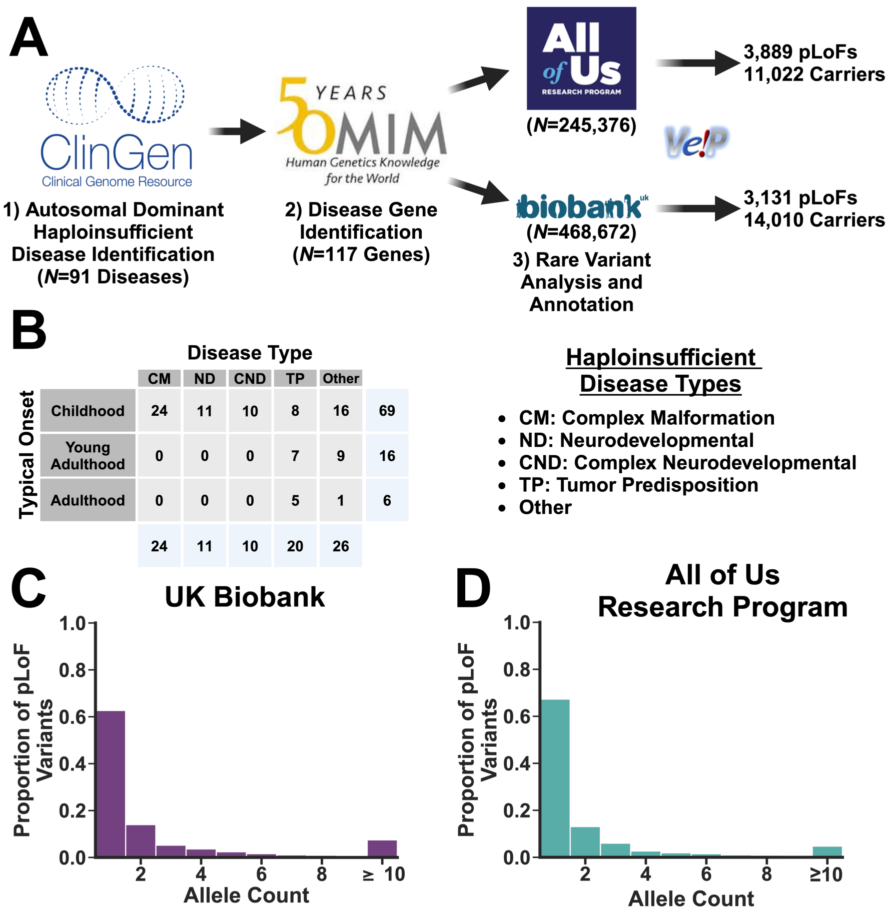

Figure 1: *Haploinsufficient Disease and pLoF Identification.* (A): Schematic depicting the strategy for haploinsufficient disease identification and biobank sequence processing. (B) Summary of the 91 haploinsufficient diseases investigated in the analysis, stratified by their typical onset class and disease type (left). Disease type definitions are provided to the right. (C, D): The carrier count distributions for the pLoF variants identified in the UK Biobank (C) and the All of Us Research Program (D). Created using Biorender.com.

### *pLoFs Have Detectable Phenotypic Effects but with Reduced Penetrance*

We restricted our penetrance analyses to pLoFs within genes that were confidently linked to haploinsufficient disorders. Therefore, these variants should have detectable and replicable effects on disease expression. To test this, we constructed a unique comparison group for each disease by identifying biobank subjects that did not carry any rare variant (biobank-specific carrier frequency ≤ 0.1%) in their associated gene(s) (see Methods). We then compared haploinsufficient disease prevalence between these two groups. This analysis was limited to those diseases with at least 1 diagnosis in either the carriers or non-carriers across the two biobanks (*N*=28; 63 diseases either lacked structured diagnostic codes or had no diagnosed cases in either biobank). In a cross-biobank meta-analysis, the pLoF variants had a statistically significant (Bonferroni-corrected) effect on prevalence for approximately two-thirds (20/28) of the diseases (see Supplementary Dataset 4 for the full results). Moreover, the prevalence risk ratios for the significant associations (adjusted for recruitment age, birth sex, and the first 16 principal components of the genetic covariance matrix using logistic regression) were correlated across the two biobanks ($R^2$ = 0.53; $P$-value = $1.27 \times 10^{-4}$, Figure 2A).

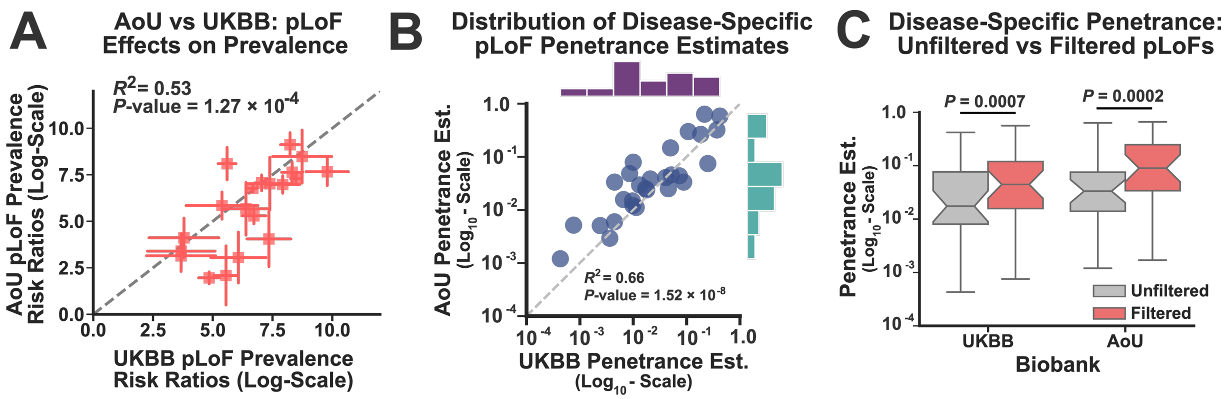

Figure 2: *pLoFs Have Detectable Phenotypic Effects but with Reduced Penetrance.* (A): Average pLoF effects on disease prevalence are compared across the two biobanks. Note, this panel only displays penetrance risk ratios for those diseases in which pLoFs had a statistically significant effect in the cross-biobank meta-analysis ($N$=20). Datapoints represent the estimated prevalence risk-ratio while bars indicate ± one standard error of this estimate. Correlation was assessed using Pearson's correlation coefficient ($R^2$). (B): Disease-specific penetrance estimates for pLoFs were computed in each biobank using diagnoses only ($N$=28). The estimates from the two biobanks were compared, and their correlation was assessed using Pearson's correlation coefficient ($R^2$). (C): Boxplots comparing the distribution of penetrance estimates before (gray) and after (red) filtering pLoFs for potential artifacts (see main text). Statistical significance was assessed using a one-sided Wilcoxon signed rank test.

These results indicate that pLoFs had consistent effects on prevalence, but this does not imply that their apparent penetrance was high. To estimate pLoF penetrance for each disease, we measured their expression using EHR-derived diagnoses, generating point estimates and 95% confidence intervals for penetrance using a simple binomial model (see Methods). Figure 2B compares the disease-specific pLoF penetrance estimates across the two biobanks (again limited to diseases with diagnostic data available in one of the two biobanks; $N$=28). Clearly, this is an imperfect estimate, as only diagnoses were used to measure disease expression. Nevertheless, the penetrance estimates across biobanks were highly correlated ($R^2$ = 0.66; $P$-value = $1.52 \times 10^{-8}$), indicating replicability but also highlighting significant disease-wide heterogeneity. Consistent with previous analyses[44], the pLoF penetrance estimates were relatively low (median across diseases = 1.7% ± median absolute deviation = 1.6% in the UKBB; 3.3% ± 2.2% in AoU).

Most studies that analyze pLoFs filter these variants to remove potential annotation artifacts[52–55], which are variants that have no molecular impact but were misannotated as LoFs by the variant prediction software. To account for such artifacts, we repeated this analysis after removing variants that impacted non-canonical transcripts (i.e. non-MANE Select[56]) and/or failed to meet a set of quality filters (assessed using the LOFTEE package[53]). In total, this filtering removed 29% and 38% of the pLoFs in the UKBB and AoU respectively (mean ± std. deviation across individual diseases: 35% ± 25% and 42% ± 25%, respectively). Restricting the analysis to the filtered variants increased the disease-specific penetrance estimates in both biobanks (Figure 2C; Wilcoxon Signed Rank Test Meta Analysis *P*-value = $9.54 \times 10^{-7}$); seven diseases even achieved pLoF penetrance estimates exceeding 20% in both datasets (ex: Autosomal Dominant Polycystic Kidney Disease and Neurofibromatosis Type 1; see Supplementary Dataset 5 for details. Note, most individual disease penetrance estimates in AoU must be suppressed due to privacy restrictions). Nevertheless, while the median penetrance estimates across diseases increased nearly 3-fold in both biobanks, they remained less than 10% (4.6% ± 3.7% in UKBB; 9.0% ± 7.2% in AoU). Therefore, variant annotation artifacts alone unlikely account for a substantial fraction of the reduced pLoF penetrance.

*Reduced Penetrance Persists after Accounting for Missing Disease Diagnoses*

Thus far, diagnoses have been used to measure disease expression and estimate penetrance. This is clearly an imperfect approach. Most haploinsufficient (*N*=63) diseases either lack the formal diagnostic data needed to estimate penetrance or simply have no diagnoses available in either biobank. Moreover, even when diagnoses are available, previous work suggests that many pathogenic variant carriers exhibit disease expression without formal diagnoses[46,57]. To overcome this limitation, we also quantified disease expression using covariate-corrected Phenotype Risk Scores (PheRS)[58,59]. These scores measure the extent to which a subject is a phenotypic outlier based on their pattern of expressed, disease-related symptoms. This analysis is possible even in the absence of formal diagnostic data (see Methods).

Figures 3A (UKBB) and 3B (AoU) display the distributions over the median PheRS estimates for each disease, which were standardized using the PheRS distributions observed in non-carriers (Methods). Although the median increase in scores among carriers relative to non-carriers was modest (0.06 in the UKBB; 0.10 in AoU), the PheRS estimates were systematically increased among carriers across the full set of diseases (Wilcoxon Signed-Rank Meta-Analysis *P*-value = $2.88 \times 10^{-11}$). We also compared the distributions of PheRS's among the carriers and non-carriers of the individual diseases, using one-sided Brunner-Munzel non-parametric statistical hypothesis tests to assess significance. Multiple diseases achieved dataset-wide (14 of 89 diseases with PheRS information available in both biobanks) or at least uncorrected (37 of 89) statistical significance in a cross-biobank meta-analysis (see Supplementary Dataset 6 for the full set of results). Therefore, the pLoF variants were systematically associated with increased symptom expression for many diseases, at least when compared to a set of biobank-derived non-carriers. However, the increase in scores among the carriers was relatively modest for most except for a few outliers (see Figures 3A and 3B).

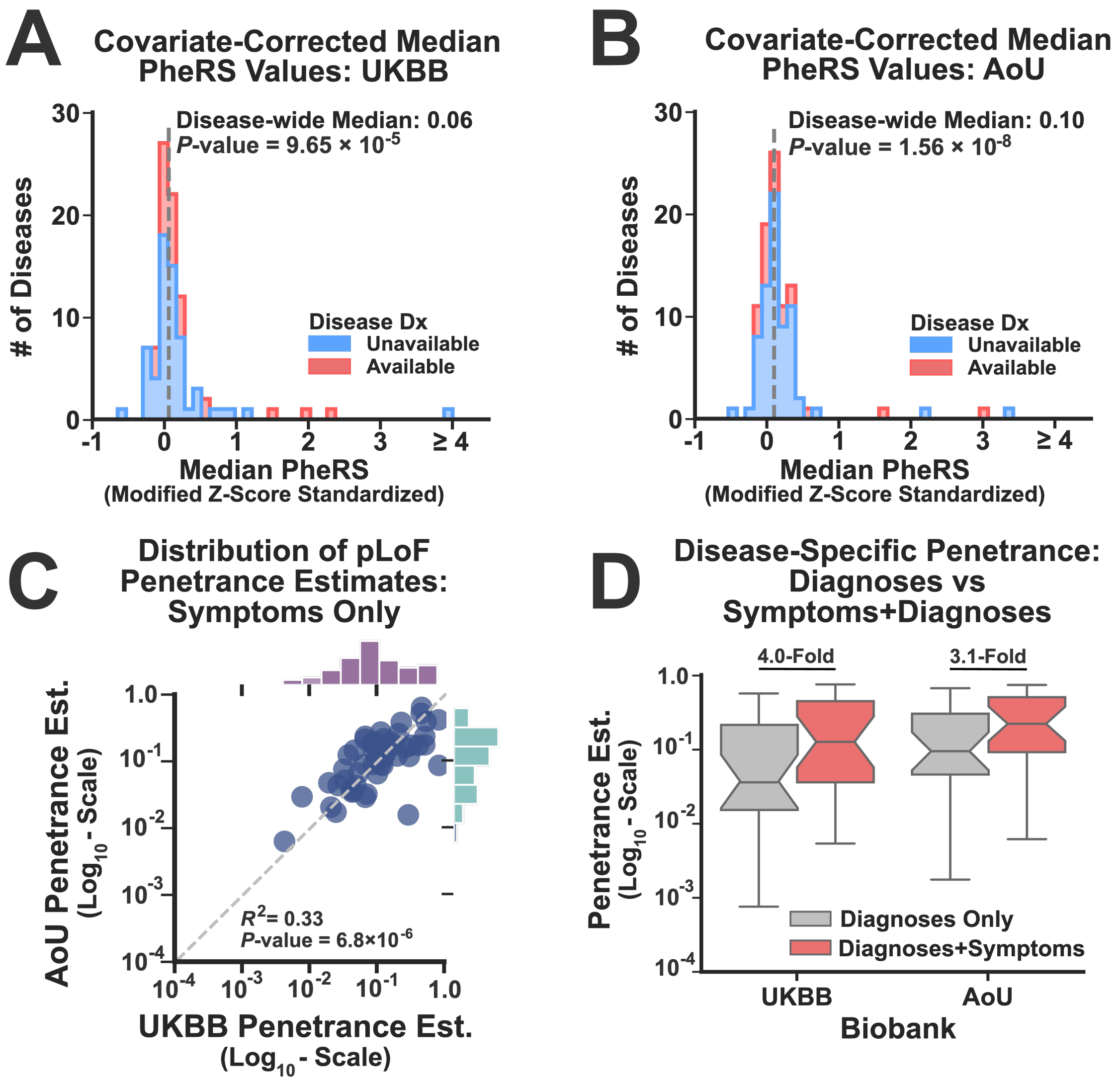

Figure 3: *Reduced Penetrance Persists after Accounting for Missing Disease Diagnoses.* (A, B): The median Phenotype Risk Score (PheRS) for each disease was computed using the pLoF carriers and standardized using the scores observed in the non-carriers. These histograms display the standardized, disease-specific median PheRS scores for the UKBB (B; N=90 diseases with PheRS information available) and AoU (C; N=90). Significance was assessed using a one-sided Wilcoxon signed rank test. (*C*) For those diseases without diagnostic data (N=51), the symptom-driven, disease-specific penetrance estimates for the pLoF variants were compared across the two biobanks. Correlation was assessed using Pearson's method ($R^2$). (D): For diseases with symptoms and diagnoses available in both biobanks (N=25), the boxplots compare the disease-specific penetrance estimates before (gray) and after (red) incorporating the symptom-driven expression measurements. The fold-increase in the median that occurred after including the symptom-driven measurements is depicted at the top of the boxplots.

Unfortunately, the PheRS method cannot directly be used to produce penetrance estimates. It is a continuous score whose range varies with the number symptoms assigned to each disease. Moreover, the threshold for an elevated PheRS depends upon the background frequency of disease-associated symptoms in the general population of non-carriers. Common symptoms (examples: fatigue, muscle pain, proteinuria, etc.) will yield non-zero PheRS's but may not be indicative of pLoF expression. To overcome these issues, we developed a statistical model that converted PheRS's into disease expression probabilities by comparing the frequency of diagnosed symptoms among pLoF carriers to a background distribution. If a carrier's diagnosed symptoms were unlikely according to this background distribution but were consistent with the symptoms expressed by other pLoF carriers, then they were assigned a high probability of disease expression (see Methods). This model generated a unique expression probability estimate for each pLoF carrier, which could then be incorporated into our penetrance estimates (see Methods). In Figure 3C, we display the distributions of symptom-driven penetrance estimates for the 51 haploinsufficient diseases that lacked diagnostic data but shared documented symptoms across the two biobanks. For these diseases, the purely biobank-specific, symptom-driven penetrance estimates were correlated ($R^2 = 0.33$; $P$-value = $6.8 \times 10^{-6}$), indicating that these measurements were capturing consistent phenotypic effects as well as the penetrance heterogeneity across diseases (see Supplementary Information for additional information).

Overall, incorporating symptom-driven expression measurements increased our sample size and improved our ability to detect haploinsufficient disease expression in the biobanks. However, for diseases with phenotype data that was shared by both datasets ($N$=76), pLoF penetrance remained incomplete for all diseases (median penetrance estimates: 8.4% ± 6.1% and 14.1% ± 8.6% in the UKBB and AoU respectively). For those diseases with both diagnoses and symptom-driven expression measurements available in both biobanks ($N$=25; 3 diseases from Figure 2 did not share symptom data across biobanks), including symptoms increased the penetrance substantially (12.5% ± 11% and 22.0% ± 17.4% penetrance for the UKBB and AoU, respectively, Figure 3D). However, most pLoF carriers still did not display evidence for disease expression. The full set of symptom-driven penetrance estimates (which include diagnosis-derived measurements if available) are provided in Supplementary Dataset 7.

*Incomplete pLoF Penetrance Persists After Accounting for Gaps in EHR Data Coverage*

Another factor that could negatively impact penetrance estimation is the incomplete lifetime coverage of the EHR data in biobanks[42,46]. More specifically, these datasets capture only a fraction of their subjects' lifespans, and the data coverage for individual participants can vary widely (see Supplementary Figure 1). Missing clinical data, which can occur during any lifetime interval (e.g. childhood, late adulthood, etc.), can allow a subject to appear asymptomatic and thus bias penetrance estimates downward. To address this issue, we developed a statistical filtering strategy to remove pLoF carriers whose EHR data coverage was insufficient to accurately measure disease expression (see Methods for details). This filtering removed 17% and 35% of the pLoF carriers from the UKBB and AoU respectively, with more extensive filtering occurring in the latter biobank due to its tendency to have more limited data coverage.

Figure 4A depicts the change in pLoF penetrance estimates (using both diagnoses and symptoms if available, otherwise symptoms only) for the pLoFs within both datasets after removing subjects with low clinical data coverage. Penetrance increased systematically but modestly in both biobanks (fold-increase in median penetrance: 1.9 and 2.0-fold for the UKBB and AoU, respectively; Wilcoxon Signed Rank Test Meta Analysis *P*-value < $2.2\times10^{-16}$). After all three of our data processing steps (annotation artifact removal, accounting for missed diagnoses, and filtering subjects with incomplete clinical data, see Figure 4B), the median penetrance for diseases with diagnoses and symptoms available in both biobanks (*N*=25) increased 10.9-fold (1.7% ± 1.6% vs 18.5% ± 16.7%) and 6.1-fold (3.8% ± 3.2% vs 23.3% ± 19.1%) in the UKBB and AoU respectively (see Supplementary Dataset 8 for the full set of estimates). However, no haploinsufficient disease achieved a pLoF penetrance estimate exceeding 80% in either dataset. Moreover, the median estimates for childhood-onset disorders remained much lower than expected (9.7% ± 6.2% and 16.7% ± 8.9% in the UKBB and AoU, respectively), particularly when compared to the pLoF penetrance for young adult-onset diseases (26.9% ± 22.3% and 41.5% ± 20.0% respectively). Collectively, this suggests that factors other than annotation artifacts and missing data are driving the incomplete pLoF penetrance observed in biobanks.

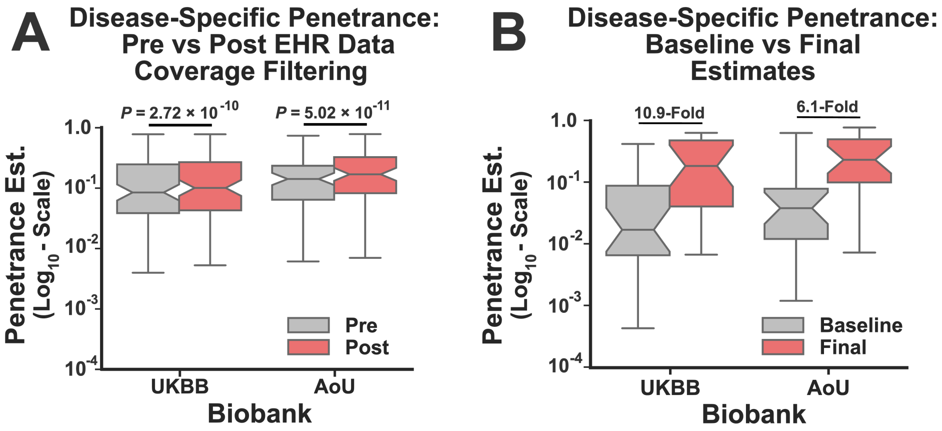

*Figure 4: Incomplete pLoF Penetrance Persists After Accounting for Gaps in EHR Data Coverage*. (A) These boxplots depict the disease-specific penetrance estimates before (gray) and after (red) removing pLoF carriers with insufficient clinical data coverage. Statistical significance was assessed using a one-sided Wilcoxon signed rank test (*N*=76). (B): For diseases with symptoms and diagnoses available in both biobanks (*N*=25), the boxplots compare the disease-specific penetrance estimates before (gray) and after (red) completing all three of our processing steps. The fold-increase in the median penetrance estimates is shown at the top.

### *Variant-Specific Genomic Features Predict Widespread Reduced pLoF Penetrance*

After removing annotation artifacts, accounting for missed diagnoses, and correcting for incomplete EHR coverage, pLoF penetrance estimates increased systematically. However, reduced penetrance remained common. None of the filters we employed were perfect, and deficiencies in our processing steps could certainly account for some additional fraction of the incomplete penetrance. However, we also considered the possibility that some pLoFs may simply have intrinsically reduced penetrance. More specifically, it is commonly assumed that variants predicted to cause loss-of-function fully disrupt allelic activity, implying that they are equivalent in terms of their molecular impact (i.e. "complete" loss of function). This may not be the case. Some may have deleterious effects but still harbor residual or even near-complete allelic activity. For example, incomplete non-sense mediated decay escape[60,61] could allow for the partial expression of a transcript impacted by a stop-gain variant. Such "leaky" expression could in turn cause the apparently reduced penetrance that we observed in the biobanks. This is particularly true for diseases driven by dosage sensitivity, where variability in gene expression and/or activity levels may lead to heterogenous phenotypic expression[62]. Detecting such effects for individual pLoFs using biobank data is challenging. However, by comparing pLoF expression rates to genomic features that may correlate with "leaky" allelic activity, we hypothesized that we could generate systematic evidence for variant-specific incomplete penetrance.

To do so, we conducted a cross-biobank machine learning analysis (illustrated in Figure 5A) designed to predict pLoF penetrance from genomic features that either directly or indirectly capture incomplete loss-of-function. First, we identified cohorts of pLoF variant carriers in both biobanks, filtering out those with inadequate clinical data coverage (see above and Methods). We then measured disease expression among pLoF carriers using symptoms (and diagnoses, if available), producing scores that quantified their expression risk (ranging from 0 to 1, see Methods). Each pLoF was assigned to one of three classes (stop gain, frameshift, or splice change), after which it was annotated with a set of genomic features (6 unique features for stop gain variants, 6 for frameshifts, and 13 for splice changes) predicted to correlate with incomplete loss-of-function (examples are provided in Figure 5A, see Supplementary Information for more details). Finally, using the UKBB exclusively, we trained machine learning models[63] (one for each pLoF class; see Methods) to predict the pLoF disease expression scores directly from these features. The predictive performance of these models was then independently evaluated in AoU. If our hypothesis were correct, the models should be predictive of pLoF penetrance in this validation dataset, even for variants with prior evidence for pathogenicity according to diagnostic testing laboratories.

Figure 5B summarizes the performance of the pLoF penetrance prediction models in the AoU validation dataset. Each point on this curve represents a distinct, model-derived penetrance prediction scoring threshold, sorted from highest to lowest. The y-axis depicts the average penetrance for the pLoFs with prediction scores exceeding this threshold, while the x-axis displays the fraction of all penetrant pLoFs (weighted by their disease expression scores) captured by these thresholds (equivalent to recall). Perfect predictive performance is illustrated by the dashed red line (100% average penetrance with 100% recall), while the gray dashed line indicates the baseline penetrance of the pLoFs. If the prediction models are effective, then the average penetrance of the variants should increase systematically as the scoring threshold becomes more selective. Based on the results in Figure 5B, the machine learning penetrance prediction models clearly performed better than baseline (increase in pLoF penetrance from baseline averaged over all possible scoring thresholds[64] = 11.9%; bootstrapped $P$-value $< 1.0\times10^{-4}$), with penetrance exceeding 50% at selective thresholds. For reference, we also depict the performance of two simpler methods for identifying highly penetrant pLoFs: the annotation artifact filter employed previously (pLoFs in MANE Select transcripts[56] with a high confidence LOFTEE flag[53]) and variants with non-conflicting pathogenic/likely-pathogenic (P/LP) annotations in ClinVar[47]. Averaged over all possible scoring thresholds (while weighting by recall, see Methods), the machine learning models identified pLoFs with significantly higher average penetrance than either (22.7% vs 12.1% or 12.6% respectively; bootstrapped $P$-value < $1.0\times10^{-4}$). Moreover, the models even outperformed these simple filters at the precise thresholds that matched their recall (17.7% vs 13.0% and 20.7% vs 14.8% for MANE Select/LOFTEE and P/LP respectively, see Figure 5B).

In Figure 5C, the performance of the penetrance prediction models is stratified by pLoF class (stop gain, frameshift, and splice change) and compared to the baseline penetrance of each (dotted lines). The models outperformed the baseline penetrance estimates for all three classes (bootstrapped $P$-values < $1.0\times10^{-4}$), but the penetrance for splice change variants was only marginally increased by the model, even at selective scoring thresholds (avg. penetrance increase from baseline: 5.1%). This suggests that this class of variants may be associated with the highest rates of incomplete penetrance. Despite the variability in predictive performance, feature importance analysis[65] indicated that a consistent set of variant-intrinsic genomic features contributed to the performance each model, with an omnibus score of deleteriousness (CADD[66]) and relative amino acid position[67] being among the most predictive features for all three (see Supplementary Figures 5-7). That said, there were also some class-specific features that also contributed substantially to predictive performance (ex: features predicting NMD escape for stop-gains[60], see Supplementary Figure 5), suggesting that penetrance prediction could be improved by incorporating additional class-specific features.

Figure 5D again displays the performance of the penetrance prediction models, except now the results are separated by disease-onset. Even pLoFs in adult-onset, haploinsufficient disease genes can be stratified to maximize penetrance (avg. penetrance increase from baseline = 10.6%; bootstrapped $P$-value < $1.0\times10^{-4}$). However, they have the lowest average penetrance overall, likely because reduced penetrance is more common for these diseases coupled with the fact their expressivity is the hardest to measure in biobanks due to right censoring. Figure 5E displays the penetrance prediction model performance curves exclusively for variants annotated as P/LP in ClinVar (non-conflicting), again stratified by disease onset. Overall, the machine learning models remained strongly predictive of penetrance for this class of variants (avg. penetrance increase from baseline = 20.0%; bootstrapped $P$-value < $1.0\times10^{-4}$), with the most stringently filtered pLoFs now approaching near complete penetrance for all three onset classes. Based on these results, it is highly unlikely that incomplete clinical data is accounting for much of the apparent reduction in penetrance, as missing data should not correlate with variant-specific genomic features independently of their effects on disease expression. Instead, there is a much more likely explanation for this result[42]: many clinically-annotated P/LP variants have reduced (or even near-zero) penetrance, which can be predicted from genomic features.

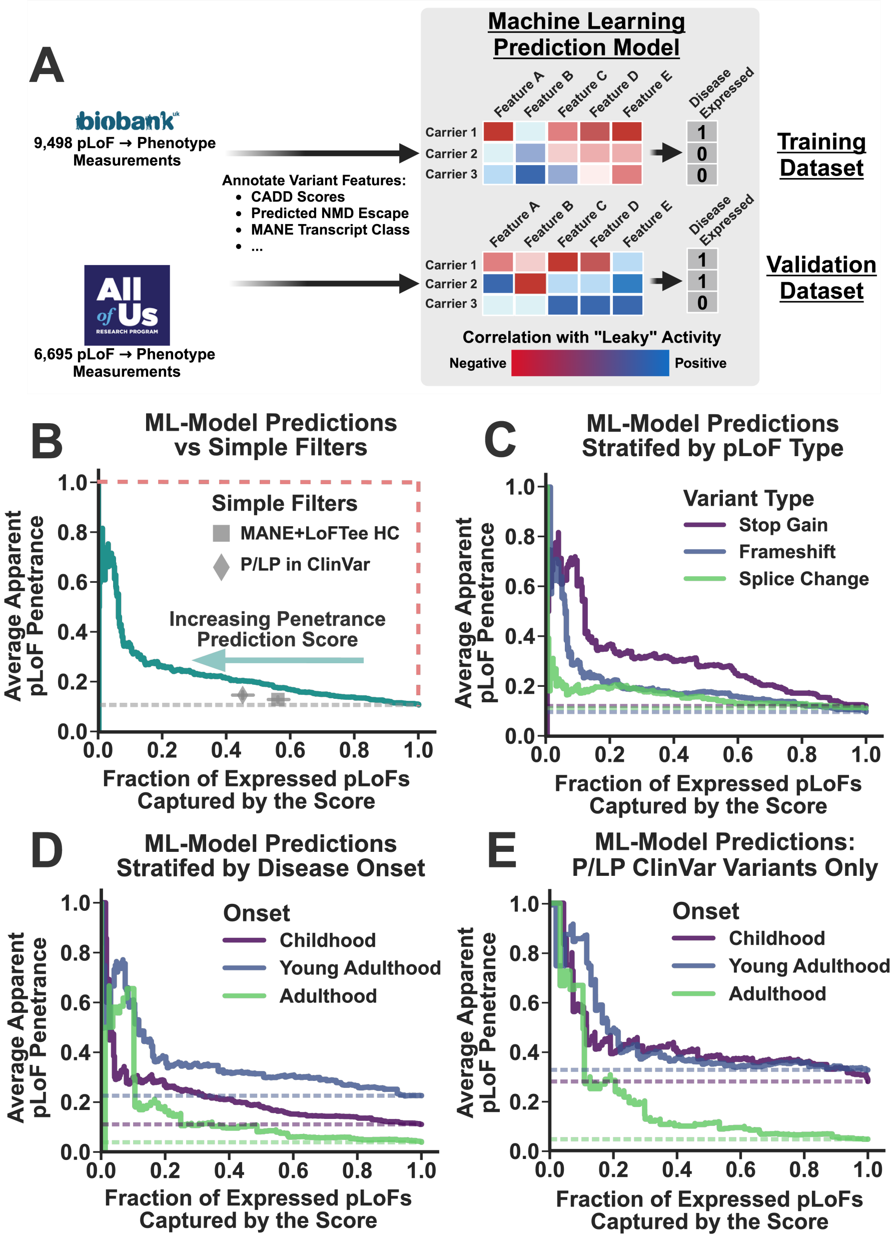

*Figure 5: Variant-Specific Genomic Features Predict Widespread Reduced pLoF Penetrance*
(A): Schematic illustrating our cross-biobank machine learning analysis to investigate reduced pLoF penetrance. (B): This panel displays the tradeoff between average apparent pLoF penetrance (y-axis) and the fraction of expressed pLoFs recalled (x-axis) when using different model-derived penetrance prediction scores to filter variants. The performance of a simple filter (MANE Select+High-Confidence LOFTEE flag) is shown as a gray square, while a filter that only includes variants with non-conflicting pathogenic/likely-pathogenic (P/LP) annotations in ClinVar is shown as a gray diamond. All error bars represent bootstrapped 95% confidence intervals. The gray dotted line indicates the baseline penetrance rate, and the red dotted line indicates the performance of a perfect classifier (C, D): These panels depict the same results from (B), except now pLoFs are stratified by variant type (C) or typical disease onset (D). Dotted lines indicate the baseline penetrance for each variant or onset class. (E): This panel depicts the penetrance prediction model performance curves for those pLoFs with non-conflicting P/LP annotations in ClinVar, again stratified by disease onset. Created using Biorender.com.

## Discussion

Exome and genome sequencing have revolutionized the field of clinical genetics[68]. More rare disease patients are being diagnosed using these technologies, and this has improved our ability to provide timely counseling and treatment to the affected individuals and their families. As a result, there is growing interest in applying broad genomic testing to asymptomatic individuals[7–18], with the goal of identifying individuals at risk for a Mendelian disease prior to symptom onset. Theoretically, this could lead to better clinical outcomes through earlier diagnosis, surveillance and management. For prognostic genomic testing to succeed, it should at least have a quantifiable positive predictive value[19–21], a statistic that directly depends on penetrance. Currently, penetrance is almost universally unknown except for a handful of unusually frequent, deleterious variants. As a result, Mendelian disease risk assessments will be imprecise for most asymptomatic carriers. This may have a limited impact on patient outcomes in many settings. However, the clinical decisions made using genomic testing will be life altering in some cases, and without penetrance information, such interventions may be unnecessarily applied to low-risk carriers.

In this study, we investigated the penetrance of one of the simplest classes of clinically relevant genetic variants: predicted loss-of-function variants (pLoFs) in haploinsufficient (autosomal dominant) disease genes. Consistent with prior analyses[43,44,57], we found that the apparent penetrance of these variants in biobanks was generally low. Accounting for variant annotation artifacts and missing clinical data increased penetrance estimates, but most pLoFs remained unexpressed. Distinguishing between high and low risk pathogenic variants represents a significant challenge for prognostic genetic testing. In diagnostic applications, detailed criteria for variant interpretation have been developed to mitigate the risk for false positive results[28,69]. These criteria cannot be directly applied to asymptomatic carriers to predict risk. Moreover, even variants with prior evidence for pathogenicity based on diagnoses in symptomatic individuals had incomplete penetrance in the biobanks, suggesting that the utility of clinical annotations for penetrance prediction is limited. Alternatively, machine learning models that incorporated variant-specific genomic features like omnibus scores of deleteriousness[66], splicing predictions[70], and putative non-sense mediated decay escape[60] successfully stratified pLoFs according to their penetrance. This suggests that many pLoFs have (sometimes very) low penetrance, enabling their carriers to avoid disease expression via “leaky” or partial allelic activity.

These results imply that screening tests for and incidental findings related to haploinsufficient diseases that rely on current pLoF interpretation guidelines will have low positive predictive values. However, there are several limitations to this analysis. First, biobanks recruit subjects from adult populations, which are almost certainly depleted of severe, early-onset phenotypes. As a result, pLoF penetrance estimates derived from biobanks may be lower than those obtained using cohorts recruited at birth, although this will vary based on a disease’s age-of-onset, it’s impact on survival, and the recruitment strategies used by the biobanks. Second, our machine learning penetrance prediction models did not incorporate any gene or disease-level features. Penetrance likely varies systematically across these entities, and future work will be needed to decompose their contributions to penetrance. Finally, penetrance variability is also driven by variant-extrinsic features, such as polygenic background and environmental modifiers, and these factors could also be incorporated into the risk prediction models for asymptomatic carriers.

Along these same lines, we limited our analysis to pLoF variants linked to 91 haploinsufficient diseases. Although they covered a range of human pathophysiology, these represent the simplest class of pathogenic variants and capture only a tiny fraction of Mendelian disease-causing mutations. That said, loss-of-function is also the predominant mechanism for hundreds if not thousands of autosomal recessive diseases, and our findings likely generalize to these and other diseases that are sensitive to allelic activity and gene “dosage.” There are also many more diseases (autosomal dominant or otherwise) driven by a variety of molecular mechanisms, and it’s possible that reduced penetrance is even more pervasive for some (ex: missense variants in haploinsufficient disease genes). Finally, the machine learning models used in the current study were developed to provide evidence for widespread reduced penetrance in biobank datasets. However, their accuracy in real-world applications remains unknown. To be clinically useful, much work will be needed to build models that are replicable, calibrated, minimally biased, and broadly applicable to diverse genes, diseases, and populations.

For now, it may be possible to predict the phenotypic outcomes for some rare genotypes with near complete penetrance[71]. In addition, when variant information is combined with orthogonal data like enzymatic activity and biomarkers, the prognostic accuracy may be very high[72]. Unfortunately, such assays are available for only a tiny fraction of genetic diseases. For most, the variants themselves provide the only piece of prognostic information. Prior clinical evidence for pathogenicity may increase the positive predictive value for a particular variant, but based on the analyses presented here, prior pathogenic annotation labels are at best poorly correlated with high penetrance. Given the distinct goals of diagnostic and prognostic genetic testing, this is not unexpected. Access to high-quality outcome data for individual genotypes will certainly help, but given their intrinsically low frequency, it will likely remain difficult to estimate penetrance and predict disease outcomes in individual patients for the foreseeable future. Therefore, we suggest that caution be used when returning positive genomic findings to asymptomatic patients, including those detected incidentally during routine diagnostic testing[18]. Even with prior evidence for pathogenicity, variant-associated disease risk remains uncertain and may be quite low.

## Methods

*Haploinsufficient Disease Curation and Annotation*

We used the ClinGen[48] Database (downloaded on July 25th, 2023) to identify Mendelian disorders that have strong evidence to support haploinsufficiency as a mechanism of disease (ClinGen Dosage Haploinsufficiency Assertion Evidence Level 3). We then aligned these diseases to the Online Mendelian Inheritance in Man[49] database (downloaded on February 23, 2023) using simple string matching followed by manual curation. This yielded 91 autosomal dominant/pseudo-autosomal dominant diseases linked to 117 genes, which were manually annotated with their typical onset (Childhood, Young Adulthood, Adulthood) and general classification (Congenital Malformation, Isolated Neurodevelopmental, Complex Neurodevelopmental, Tumor Predisposition, and Other) by a board-certified medical geneticist (author D. Blair) using clinical expertise and literature review. Afterwards, disease-specific diagnostic codes were annotated to these diseases by manually curating the terminologies[73] used by the Observational Medical Outcomes Partnership Common Data Model[74] (OMOP-CDM). Finally, the diseases were annotated with a set of Human Phenotype Ontology[75] (HPO) symptoms using the data from several ontologies, including the HPO itself (downloaded on February 21, 2023), the Disease Ontology[76] (downloaded on February 23, 2023) and OrphaNet[77] (downloaded on February 23, 2023 using the HOOM[78] module). The sequence and transcript information for each of the 117 genes was downloaded from the Ensembl[79] database (Release 109; GRCh38 assembly) using the PyEnsembl[80] package. Additional gene and transcript information (exon-intron boundaries, 5' and 3' UTRs, full coding and amino acid sequences) was downloaded using gget[81]. The 91 haploinsufficient diseases, along with their annotated information, are provided as Supplementary Dataset 1.

*Aligning HPO Symptoms to the OMOP-CDM Terminology*

To identify HPO[75] symptom expression in the EHR data, we needed to align this ontology to the structured diagnostic data available in the electronic health records of each biobank. Because both biobanks encode their clinical data using the OMOP-CDM[74], we focused on aligning the HPO symptom terminology to the structured vocabulary used by this data model[73]. Unfortunately, aligning the HPO to other medical terminologies is largely an unsolved problem that lacks a consensus regarding best practices[82]. Therefore, we created a custom alignment by building on our previous work[25] while implementing some new techniques.

First, we created an alignment map between the HPO and SNOMED-CT[83], as the latter represents the most comprehensive medical terminology available for the dissemination of EHR data. It is also fully incorporated into the concept terminology used by the OMOP-CDM. To create an HPO-to-SNOMED map, we followed the approach of McArthur et al.[84], who created a similar map between the HPO and PheCodes[85]. First, we constructed a map linking HPO to SNOMED-CT terms if they shared a common concept in the UMLS Metathesaurus[86]. Second, we used an ontology alignment algorithm (SORTA[87]) to find all SNOMED-CT terms that mapped to an HPO

term with a similarity score of ≥ 0.8 for at least 1 of their associated string pairs (both SNOMED-CT and HPO often provide multiple strings for each term). For terms with multiple aligned string pairs, we collated all the similarity scores across the different string pairs, storing both an average and maximum score.

With an HPO-to-SNOMED map in place, the HPO terms themselves could be directly aligned to the concept terminology used by the OMOP-CDM, as a map from SNOMED-CT terms to the OMOP-CDM concepts is provided by the Observational Health Data Sciences and Informatics (OHDSI) Collaborative[73,88]. However, it is important to note that one HPO term often mapped to multiple SNOMED-CT terms, which could then map to the OMOP vocabulary in multiple ways. Therefore, each HPO-OMOP alignment was often supported by multiple intermediary relationships. To summarize this phenomenon, we stored several pieces of information for each alignment that captured the quality of its supporting evidence. These included: the total number of intermediate relationships supporting the mapping, the fraction of these relationships that were supported by the UMLS, the fraction that achieved a SORTA string alignment similarity score ≥ 0.8, the average SORTA score across intermediaries, and the maximum score achieved. In total, this process generated 35,825 unique HPO-to-OMOP alignments.

Because automated alignments like this tend to be rife with spurious results, one of the authors (D. Blair) manually reviewed 500 random mappings and annotated their medical accuracy. The accuracy was unsurprisingly variable, but overall, far better than random (average precision: 0.76). To further improve accuracy, we built a simple logistic regression classifier (implemented in sklearn[89]) to predict if an HPO-OMOP alignment was accurate. The model incorporated the alignment features described above as linear predictors (noting that the maximum achieved SORTA score was incorporated as interaction term with the total number of intermediate relationships). The model was trained on the 500 manually annotated alignments prior to being applied to the full dataset. In leave-one-out 5-fold cross validation experiments, the area under the receiver operator characteristic curve for the model predictions was 0.76 (standard error: 0.017), indicating that these predictions could provide a substantial improvement to alignment accuracy. Therefore, all ≈35,000 HPO-to-OMOP alignments were scored using the prediction model, and several false positive rate (FPR) thresholds were selected for downstream filtering. The complete set of HPO-to-OMOP Concept ID alignments (along with their features, manual annotations, machine learning scores, and whether they survived various FPR filtering thresholds) are provided as Supplementary Dataset 9. Finally, we experimented with various alignment FPR thresholds in downstream analyses. Overall, PheRS enrichment among pLoF carriers was highest when using the relationships that survived a 20% FPR threshold (data not shown). Therefore, this set of alignments was used for the results reported in thes manuscript.

### *Sequence Data Quality Control, Variant Annotation, and Non-Carrier Cohort Identification*

This study utilized the exome sequence (ES) data from the UK Biobank (UKBB)[37] and the genome sequence (GS) data from the All of Us (AoU) Research Program[41] to investigate the penetrance of predicted loss-of-function (pLoF) variants in haploinsufficient disease genes. For the AoU dataset, the GS samples undergo an extensive quality control process, which ensures that samples meet several coverage and accuracy thresholds[41]. Therefore, all samples with GS data that were not flagged by AoU's quality control pipeline were analyzed in this study (*N* = 245,376). For the UKBB, less sample-level quality control was performed *a priori*. Therefore, the ES data from this biobank underwent additional quality control filters consistent with those performed in previous studies[90]. Briefly, all samples that showed evidence for genetic and self-reported sex discordance (*N* = 296), sample duplication (*N* = 56), excessive SNP array-short read sequencing genotype discordance (*N* = 513, including those that lacked array data), low read coverage for the haploinsufficient genes of interest (20x coverage at less than 90% of the base pairs; *N* = 92), and excessive missing genotypes (*N* = 329, again limited to the haploinsufficient genes of interest) were excluded from the analysis (total number of samples that failed quality control: 1,156). After excluding subjects that withdrew from the UKBB study, this dataset contained a total of 468,672 subjects with both ES and EHR data.

Following sample-level quality control filtering, variants from the exonic regions of the haploinsufficient disease genes were isolated from both datasets (performed using bcftools[91] in the UKBB and hail[92] in AoU), storing the variant genotyping calls in VCF files. Individual-level data was then stripped from these files, and the predicted molecular effect of each variant was annotated using VEP[93] (Version 110). Simultaneously, the variants were annotated with any previous interpretations documented within the ClinVar[47] database (downloaded on May 13$^{th}$, 2024). Finally, all pLoFs within these datasets were identified using the LOFTEE[53] plug-in for VEP, which also provided a flag indicating the overall confidence in this assessment (high vs low confidence; HC vs LC). Using the output from VEP, each pLoF was annotated with its most clinically significant impacted transcript[56] (MANE Select, MANE Plus Clinical, Other), and the variants were also assigned to one of three pLoF classes: stop gain, frameshift, and splice change, with other variant types (e.g. missense) excluded.

Following annotation, we returned to the VCF files that contained the individual-level genotype calls and isolated all pLoFs identified in the previous step. We then identified all carriers for each individual variant, removing those that did not meet a basic set of genotype-specific quality control filters[90]. For single nucleotide variants (SNVs), we assigned a no-call status to all carriers with a genotyping quality score < 30, sequencing depth < 7, or alternate allelic balance < 0.15. For insertion-deletion variants, we were more stringent and removed those calls with a quality score < 30, sequencing depth < 10, or alternate allelic balance < 0.2. In addition, we removed a variant from the analysis entirely if its call rate was <0.99 or if its carrier frequency in the biobank was greater than 0.1% (to remove common variants that were more likely to have low or no penetrance). For the UKBB, we also *a priori* removed those variants that achieved an average read depth <10 for more than 10% of the samples in the dataset (per recommended best practices[94]). In total, this process identified 3,131 (Supplementary Dataset 2) and 3,889 (Supplementary Dataset 3) pLoFs carried by 14,010 and 11,022 subjects in the UKBB and AoU respectively. Note, some individuals harbored multiple pLoF variants within a single gene (*N*=637 in the UKBB; *N*=304 in AoU), suggesting the possibility of sequencing artifacts versus *in cis* rescue events. Such carriers were not excluded from basic pLoF carrier frequency estimates (i.e. Figure 1) but were excluded from all other analyses. As a result, the impact of *in cis* rescue events on penetrance is a target for future work[55]. Individuals with multiple pLoFs in distinct halploinsufficient disease genes were not dropped from the analysis.

Finally, for each haploinsufficient disease, we created a unique cohort of non-carriers that were unlikely to be at risk for the disease of interest. To do so, we first identified all subjects in both biobanks that carried any rare variant (allele frequency ≤ 0.1%) in the gene(s) annotated to each disease. We then dropped these subjects from the set of all biobank subjects (along with those that had a no-call genotype at one of the detected pLoFs). This yielded a unique set of non-carriers for each disease. The total number of non-carriers available for the different diseases was variable but exceeded 280,000 and 130,000 in all instances for the UKBB and AoU respectively.

*Haploinsufficient Disease Prevalence and Penetrance Analysis*

The simplest way to measure pLoF-driven disease expression was using diagnoses. Let $\vec{D}$ denote a binary vector of length *N*, where in *N* is the number of pLoF carriers for some haploinsufficient disease of interest plus the number of non-carriers identified in the biobank. Let $D_i = 1$ denote that the *i*th subject was diagnosed with the disease of interest at least once in their EHR data, where diagnoses were identified using a set of manually annotated OMOP-CDM concept codes (see Supplementary Dataset 1). Finally, let $\vec{G}$ denote a binary vector indicating the pLoF carrier status for the *N* subjects. We estimated a biobank-specific pLoF prevalence risk ratio for each disease (on the logarithmic scale and approximated using the log-odds ratio; denoted $\gamma_{\text{pLoF}}$) using one of two approaches. For the more common diseases ($\sum_{i=1}^{N} D_i \geq 10$), we incorporated covariates into the analysis using the following log-linear model:

$$\boldsymbol{P}\left(\vec{D} \middle| \mu, \vec{G}, \gamma_{\text{pLoF}}, \boldsymbol{X}, \vec{a}\right) = F\left(\mu + \vec{G} \times \gamma_{\text{pLoF}} + \boldsymbol{X} \times \vec{a}\right), \tag{2}$$

where $F$ denotes the logistic function, $\mu$ is an intercept term, $\boldsymbol{X}$ is a matrix of covariates, and $\vec{a}$ is a vector of covariate effect size parameters. For the current study, we incorporated the following covariates into our analysis: recruitment age, birth sex, and the first 16 principal components of the genetic relatedness matrix. Model fitting was performed using Firth-penalized maximum-likelihood estimation[95], and statistical inference was conducted using a likelihood ratio test. Even with Firth penalization, model inference returned spurious results when $\vec{D}$ was extremely sparse. Therefore, for very rare diseases ($\sum_{i=1}^{N} D_i < 10$), we constructed $2 \times 2$ contingency tables from $\vec{D}$ and $\vec{G}$ and estimated the (covariate-uncorrected) pLoF prevalence risk ratio and its corresponding standard error using the statsmodels[96] software package in Python. *P*-values were computed using Fisher's exact test[97]. Finally, we performed cross-biobank meta-analyses of the pLoF prevalence risk using the Cochran-Mantel-Haenszel Test for stratified contingency tables (implemented in the statsmodels[96] package). Supplementary Dataset 4 contains a summary of the results of our disease-specific prevalence association analyses.

To estimate disease-specific pLoF apparent penetrance (denoted $\hat{\mathcal{P}}$) using diagnoses, we assumed a symmetric beta prior distribution over $\mathcal{P}$ with hyper-parameter

$\theta = 0.5$. Assuming disease diagnoses among pLoF carriers follow a Bernoulli process, the posterior distribution over $\mathcal{P}$ is:

$$\mathcal{P} \sim Beta\left(\theta + \sum_{i=1}^{N} D_i \times G_i, \theta + \sum_{i=1}^{N} (1 - D_i) \times G_i\right),$$

such that an estimator for the mean pLoF penetrance (denoted $\hat{\mathcal{P}}$) is simply:

$$\hat{\mathcal{P}} = \frac{\theta + \sum_{i=1}^{N} D_i \times G_i}{2\theta + \sum_{i=1}^{N} G_i}. \quad (3)$$

In practice, disease-specific penetrance was estimated in each biobank independently, allowing the estimates to be compared across datasets (ex: Figure 2B). Note, the prior distribution was incorporated into the analysis to smooth our penetrance estimates in the face of sparse data. For diseases with very low pLoF carrier counts, the prior can have an outsized effect. This should have no appreciable impact on our machine learning analysis but may upwardly bias absolute pLoF penetrance estimates, especially when the number of pLoF carriers is small.

*Phenotype Risk Score Analysis*

Most haploinsufficient diseases lack structured diagnostic codes that can be used to identify disease expression in EHR data. In such instances, it can be difficult to determine if a subject is in fact expressing the disease phenotype. Phenotype Risk Scores[58,59] (PheRS's) were developed to address this issue. These scores measure the extent to which a subject represents an outlier in phenotype space. Their effectiveness relies on a critical assumption: affected Mendelian disease-associated variant carriers should express constellations of symptoms that are highly atypical when compared to their non-carrier counterparts. Although this is sometimes true, it is not the case for all diseases. Moreover, non-carriers can become phenotypic outliers as well, for example, if they develop unusual complications from a common disease or multiple diseases at once. Therefore, PheRS's represent an alternative, albeit imperfect, method for measuring disease expression among pathogenic variant carriers.

Let $\vec{S}_i$ denote a binary vector of length *K*, where is *K* is the number of symptoms annotated to the haploinsufficient disease of interest (derived from multiple ontologies and aligned to structured EHR data, see above). Let $S_{i,k} = 1$ indicate that the *k*th symptom was diagnosed at least once in the *i*th subject's EHR data. Finally, let $P(\vec{S}_i|\theta)$ denote a population-wide, background probability model (defined by the parameter set $\theta$) that yields the risk of observing the symptom set $\vec{S}_i$. The Phenotype Risk Score for the *i*th subject (denoted $\text{PheRS}_i$) is given by:

$$\text{PheRS}_i = -\log\left[P\left(\vec{S}_i|\theta\right)\right]. \quad (4)$$

This formula is equivalent to the surprisal, or information content, of the diagnosed symptom set according to the model defined by $\theta$, and it provides a measurement for how unusual or atypical this set of diagnosed symptoms is. For the approach to be effective, we must define the background symptom expression probability model. Consistent with prior studies[58,59], we assumed a very simple (and somewhat unrealistic) background model for the observed symptoms sets: each of the *k* symptoms occurs independently of the others according to a Bernoulli process. Therefore,

$$P\left(S_{i,k}\middle|\theta_k\right) = \theta_k^{S_{i,k}} \times (1-\theta_k)^{1-S_{i,k}}.$$

To estimate the background model parameters, we assumed that the Mendelian disease cases were sufficiently rare in the general population such that their risk of biasing the symptom-specific parameter estimates (denoted $\hat{\theta}_k$) was negligibly low. Therefore, we estimated each symptom expression parameter independently using the maximum likelihood estimator for a Bernoulli process:

$$\hat{\theta}_k = \frac{\sum_{i=1}^{N} S_{i,k}}{N}, \tag{5}$$

where *N* denotes the total number of subjects in the biobank. After estimating this expression model, the $\text{PheRS}_i$ for each subject becomes:

$$\text{PheRS}_i = \sum_{k=1}^{K} -\log\left(\hat{\theta}_k\right) \times S_{i,k} - \log\left(1-\hat{\theta}_k\right) \times \left(1-S_{i,k}\right). \tag{6}$$

In practice, we further adjusted the raw PheRS's for confounding covariates (recruitment age, birth sex, and the first 16 components of the genetic relatedness matrix) using ordinary least squares regression.

After computing the covariate-adjusted, disease-specific PheRS's for every subject in both biobanks, we compared the distributions of these scores among pLoF carriers to those of their non-carrier counterparts. To asses statistical significance, we used a one-sided Brunner-Munzel Non-Parametric Hypothesis Test (implemented in scipy[97]), which evaluated the alternative hypothesis that the PheRS's observed in the pLoF carriers were stochastically greater than those observed in non-carriers. A fixed effects meta-analysis was used to assess cross-biobank significance. Finally, for the histograms in Figures 2B and 2C, the median PheRS's in the pLoF carriers were converted to modified Z-scores using the medians and median absolute deviations estimated among the non-carries. The complete set of PheRS results for all diseases are provided in Supplementary Dataset 6.

*Measuring Symptom-Driven Disease Expression*

PheRS's can suggest that a subject is a phenotypic outlier, but these scores do not necessarily provide an accurate measurement of whether a Mendelian disease is being expressed or not. For example, consider autosomal dominant polycystic kidney disease (ADPKD). Clearly, a pLoF carrier who has bilateral renal cysts complicated by chronic kidney disease is expressing the phenotype, but what if a carrier only experiences proteinuria? Proteinuria is certainly a symptom of ADPKD, so this carrier's PheRS score will be greater than 0. But proteinuria is an extremely common symptom in the general population. Therefore, just because an ADPKD pLoF carrier experiences proteinuria at some point in their life doesn't mean that they are expressing ADPKD.

To overcome this issue, we formulated the following probability model to measure symptom-driven disease expression among the pLoF carriers. Rather than relying on disease diagnoses, the model uses the pattern of symptoms documented within the EHR data to estimate disease expression risk. Disease diagnoses were specifically excluded from the set of possible symptoms. Therefore, this approach represents an orthogonal method for measuring haploinsufficient disease expression using EHR data. Consistent with the PheRS approach, let $P(\vec{S}_i|\theta)$ denote the probability that a set of symptoms $\vec{S}_i$ is being expressed according to some general background distribution. In addition, let $P(\vec{S}_i|\delta)$ denote the probability that this symptom set is instead expressed by an affected haploinsufficient disease variant carrier (where the parameter set $\delta$ defines the expression model). Finally, let $E_i = 1$ indicate that the disease of interest was expressed in the *i*th carrier. The probability of disease expression is given by:

$$P(E_i = 1|\vec{S}_i, \delta, \theta, \pi) = \frac{\pi \times P(\vec{S}_i|\delta)}{(1-\pi) \times P(\vec{S}_i|\theta) + \pi \times P(\vec{S}_i|\delta)}, \quad (7)$$

where $\pi$ is the prior probability of disease expression among all carriers. An illustration of this model is provided in Supplementary Figure 2.

Ultimately, our goal in specifying this model was to estimate $P(E_i = 1|\vec{S}_i, \hat{\delta}, \hat{\theta}, \hat{\pi})$, which is the probability of disease expression in the *i*th pLoF carrier conditional on their observed symptoms (denoted $\vec{S}_i$) and the inferred expression model parameters (denoted $\hat{\delta}$, $\hat{\theta}$, and $\hat{\pi}$). Our procedure for model inference and subsequent expression probability estimation is described in detail in the Supplementary Information. Following estimation, we directly incorporated these symptom-driven expression measurements into our estimates of disease-specific apparent penetrance using the following formula:

$$\hat{\mathcal{P}} = \frac{\theta + \sum_{i=1}^{N} \max[D_i, P(E_i = 1|\vec{S}_i, \hat{\delta}, \hat{\theta}, \hat{\pi})] \times G_i}{2\theta + \sum_{i=1}^{N} G_i}, \quad (8)$$

where $\max[A, B]$ simply returns the maximum of $A$ and $B$. Note, when a disease lacked EHR-derived diagnostic data, $D_i$ was simply set to 0 for the relevant pLoF carriers.

*Strategy for Removing pLoF Carriers with Incomplete Clinical Data Coverage*

The EHR can provide an incomplete representation of a patient's disease expression. Subjects are enrolled into biobank cohorts well into adulthood, and there is no guarantee that the records captured by the study represent their complete clinical history. Moreover, the clinical trajectories for many subjects are right-censored, meaning that they have yet to reach the target endpoint for the analysis of interest (in this case, death or disease expression). These gaps in clinical data coverage could account for much of the incomplete penetrance that we observed. Therefore, we devised a method to flag and remove subjects from our analysis whose clinical data coverage was insufficient for reliable measurements of disease expression.

Let $\vec{A}$ denote a binary vector of asymptomatic indicators, where $A_i = 1$ indicates that the *i*th pLoF carrier had no evidence for disease expression based on disease-specific diagnostic code(s) and/or documented symptoms (i.e. had no disease-relevant diagnoses or symptoms). Moreover, let $\boldsymbol{W}$ denote a matrix of clinical data coverage statistics. For this analysis, we used the following four statistics to define data coverage: Age at First Clinical Encounter, Age at Recruitment, Total Number of Documented Clinical Encounters, and Age at Last Clinical Encounter (see Supplementary Figure 1 for their distributions). These four statistics provided a basic summary of a patient's interaction with the healthcare system, at least according to the information in the biobanks. Finally, let $\vec{b}$ denote a vector of coverage statistic effect size parameters. We modeled the probability of phenotype *non*-expression (i.e. asymptomatic status) conditional on clinical data coverage using the following log-linear model:

$$\boldsymbol{P}\left(\vec{\mathbf{A}} \middle| \mu, \boldsymbol{W}, \vec{b}\right) = F\left(\mu + \boldsymbol{W} \times \vec{b}\right)$$

where $F$ denotes the logistic function and $\mu$ is the intercept term. Because different diseases will have different coverage requirements (depending on their typical onset, length of observation time needed to document symptoms, etc), we fit three versions of this model in both biobanks by grouping diseases based on their typical onset (childhood, young adulthood, and adulthood, see Supplementary Dataset 1).

More specifically, each model was repeatedly fit in both biobanks using leave-one-out 5-fold cross validation (model fitting was performed using the `LogisticRegression` function available in sklearn[89] with default hyperparameters). For each iteration, 80% of the onset-grouped pLoF carriers were used to estimate the parameters for the disease non-expression model. The remaining 20% were used for validation. Model performance was assessed in the validation subset using the area under the receiver operating characteristic curve (see Supplementary Figure 3 for results). To flag pLoF carriers with insufficient clinical data, we identified the 5% false positive rate threshold in each validation subset. If a subject in a validation subset had a non-expression probability that exceeded this threshold, they were deemed to have insufficient data for disease expression measurement and were flagged for removal in downstream analyses.

### *Predicting pLoF Penetrance using Variant-Specific Features*

We hypothesized that some pLoF variants have intrinsically low penetrance, which is driven by residual allelic activity. If true, then it should be possible to stratify pLoFs according to their penetrance risk using genomic features that correlate with incomplete loss-of-function. To test this, we developed machine learning models that predict pLoF penetrance risk given a set of genomic features that may correlate with residual allelic activity. Let $\vec{V_i}$ denote the vector of genomic features that characterize the penetrance risk for the pLoF carried by the *i*th subject. Examples of such features include its relative position within the amino acid sequence[67], the probability that it disrupts splicing[70], or an omnibus score summarizing its "deleteriousness"[66]. The goal of this analysis is to predict the disease expression risk for the *i*th pLoF carrier using these features:

$$\mathcal{P}_i = \mathcal{F}(\vec{V}_i; \theta), \tag{9}$$

where $\mathcal{P}_i$ is the disease expression score in the *i*th subject (i.e. $\mathcal{P}_i = \max\left[D_i, P\left(E_i = 1 \middle| \vec{S}_i, \hat{\delta}, \hat{\theta}, \hat{\pi}\right)\right]$, see Eqn. 8) and $\mathcal{F}$ is some function that maps the vector $\vec{V}_i$ onto disease expression probability space (via a parameter set $\theta$). Practically, several different models can accomplish this goal. For this study, we constructed $\mathcal{F}$ using the random forest algorithm[63] implemented in `sklearn`[89], which builds predictive models via an ensemble of individual decision trees. Note, we also considered simpler methods for constructing $\mathcal{F}$ (i.e. penalized logistic regression) but found that they performed systematically worse than this ensemble learning strategy (see Supplementary Figure 4), likely due to the latter's ability to capture non-linear effects.

Different types of pLoF variants have distinct features that likely impact their residual allelic activity and thus risk for expression[54]. Therefore, distinct penetrance prediction models were constructed for each of the three variant types analyzed in this study (stop gains, frameshifts, and splice changes). The features incorporated into each of our random forest prediction models are described in detail in the Supplementary Information. Model inference was performed by minimizing the logarithmic loss function of the prediction model when applied to a training cohort of carriers (training algorithm hyperparameters: min_samples_leaf=5, min_samples_split=10, n_estimators=500). Training was performed exclusively within the UKBB using a subset of pLoF carriers whose disease expression measurements were binarized to simplify inference (see Supplementary Information for details).

After fitting the machine learning models in the UKBB, they were independently validated within AoU. Model performance was assessed by ranking pLoFs according to their penetrance prediction scores (largest-to-smallest). Afterward, we computed the average penetrance (denoted as $\hat{\mathcal{E}}_s$ for the *s*th scoring threshold) and the fraction expressed pLoFs (weighted by their expression scores) captured each threshold (equivalent to recall, denoted $\mathcal{R}_s$) according to the following two formulas:

$$\hat{\mathcal{E}}_s = \frac{\sum_{i=1}^{N} \max\left[D_i, P\left(E_i = 1 \middle| \vec{S}_i, \hat{\delta}, \hat{\theta}, \hat{\pi}\right)\right] \times \mathbf{1}\left[\mathcal{F}(\vec{V}_i; \theta) \geq s\right]}{\sum_{i=1}^{N} \mathbf{1}\left[\mathcal{F}(\vec{V}_i; \theta) \geq s\right]}$$

$$\mathcal{R}_s = \frac{\sum_{i=1}^{N} \max\left[D_i, P\left(E_i = 1 \middle| \vec{S}_i, \hat{\delta}, \hat{\theta}, \hat{\pi}\right)\right] \times \mathbf{1}\left[\mathcal{F}\left(\vec{V}_i; \theta\right) \geq s\right]}{\sum_{i=1}^{N} \max\left[D_i, P\left(E_i = 1 \middle| \vec{S}_i, \hat{\delta}, \hat{\theta}, \hat{\pi}\right)\right]}$$

where $\mathbf{1}\left[\mathcal{F}\left(\vec{V}_i; \theta\right) \geq s\right]$ is an indicator function that returns 1 if the score of the *i*th variant is greater than the *s*th threshold. In the machine learning literature, this is equivalent to constructing a precision-recall curve. To measure global model performance, we computed the average penetrance score, which integrates the average penetrance values over all scoring thresholds while weighting them by the change in recall from the previous threshold:

$$\text{Avg. Penetrance Score} = \sum_{s=1}^{S} \widehat{\mathcal{E}_s} \times (\mathcal{R}_s - \mathcal{R}_{s-1}),$$

where $\{s = 1 \ldots S\}$ denotes the $S$ unique prediction scoring thresholds (sorted from largest-to-smallest). This measurement is equivalent to the average precision score, a statistic used by the machine learning community to summarize the performance of prediction models based on their precision-recall curves[64]. Statistical inference for the average penetrance scores (i.e. hypothesis testing and 95% confidence intervals) was completed using bootstrapped resampling[98]. To compare machine learning model performance to the simple filters (MANE Select+High-Confidence LOFTEE Flag or P/LP in ClinVar), the average penetrance score for each filter was computed by treating them as prediction models with only two output values (1: variant passes filter; 0: variant does not pass filter). Note, prediction model feature importance was evaluated within the AoU validation dataset (using Shapley statistics[65]), the results of which are summarized in Supplementary Figures 5-7.

Given that the UKBB and AoU were recruited from the populations of two different countries, the risk that the two datasets contained overlapping individuals (i.e. validation dataset contamination) was very low. However, the two biobanks did contain redundant (variants that occurred multiple times in each biobank) and shared (variants that occurred in both biobanks; *N*=773) pLoFs. Neither were excluded from the training or validation analyses given that they were carried by distinct individuals with conditionally independent phenotypic outcomes. Although this could limit model generalizability, it does not detract from the primary aim of the analysis, which was to demonstrate that pLoFs have intrinsically reduced penetrance. Moreover, Supplementary Table 1 provides a direct comparison of machine learning model performance for all variants and those that were unique to the AoU dataset. The difference in model performance on these two variant sets was minimal (average penetrance score = 22.7% for all variants versus 21.3% for variants unique to AoU).

### *Additional Statistical Methods*

Unless otherwise noted, the statistical analyses described in the main text and/or figure legends were performed using the implementations (sometimes with slight modification) available in the following Python packages: scipy[97], sklearn[89], statsmodels[96], and pandas[99]. Bootstrapped hypothesis testing was performed by generating empiric distributions for the target parameter estimates using re-sampling with replacement (10,000 re-samples for all tests). Meta-analyses for the different tests were performed using a fixed-effects model based on a standard normal approximation to their test statistics[100].

## Data Availability

The genomic and electronic health data used for this analysis are publicly available but have strict data use agreements. The process for obtaining access to these biobanks can be found on their respective websites: https://www.researchallofus.org/register/ (All of Us Research Program) and https://www.ukbiobank.ac.uk/enable-your-research/register (UK Biobank). Haploinsufficient disease annotations are provided in Supplementary Dataset 1. The custom HPO-to-OMOP concept alignments generated in this study are provided as Supplementary Dataset 9. All other databases used in this analysis are freely available in the public domain.

## Software Availability

All software packages used to conduct this study are open source and available in the public domain. Phenotype Risk Scores (PheRSs) and symptom-driven disease expression estimates were generated using the SymptomSetModel Python package, which was specifically constructed for this study (https://github.com/daverblair/SymptomSetModel). The scripts used for the analyses described in the paper are also available via github[101].

## Acknowledgements

The All of Us Research Program is supported by the National Institutes of Health, Office of the Director: Regional Medical Centers: 1 OT2 OD026549; 1 OT2 OD026554; 1 OT2 OD026557; 1 OT2 OD026556; 1 OT2 OD026550; 1 OT2 OD 026552; 1 OT2 OD026553; 1 OT2 OD026548; 1 OT2 OD026551; 1 OT2 OD026555; IAA #: AOD 16037; Federally Qualified Health Centers: HHSN 263201600085U; Data and Research Center: 5 U2C OD023196; Biobank: 1 U24 OD023121; The Participant Center: U24 OD023176; Participant Technology Systems Center: 1 U24 OD023163; Communications and Engagement: 3 OT2 OD023205; 3 OT2 OD023206; and Community Partners: 1 OT2 OD025277; 3 OT2 OD025315; 1 OT2 OD025337; 1 OT2 OD025276. In addition, the All of Us Research Program would not be possible without the partnership of its participants.

This research was conducted using the UK Biobank Resource under Application Number 99922, which relies on data provided by patients and collected by the NHS as part of their care and support. We are extremely grateful to the participants of the UK Biobank, without whom this research would not have been possible.

This work was supported by grants from the National, Heart, Lung and Blood Institute (K38HL164956) and the George Banks and Sarah Ellen Huntington Memorial Fund.
