# Supplementary material for: Dissecting the Reduced Penetrance of Putative Loss-of-Function Variants in Population-Scale Biobanks
Source: medRxiv. 2024 Oct 7:2024.09.23.24314008. Preprint. [Version 2] doi: 10.1101/2024.09.23.24314008 (PMC11469360; doi:10.1101/2024.09.23.24314008)
Supplement: Supplementary file 1 [file NIHPP2024.09.23.24314008V2-supplement-1.pdf]

medRxiv preprint doi: https://doi.org/10.1101/2024.09.23.24314008; this version posted October 7, 2024. The copyright holder for this preprint (which was not certified by peer review) is the author/funder, who has granted medRxiv a license to display the preprint in perpetuity.
It is made available under a CC-BY 4.0 International license .

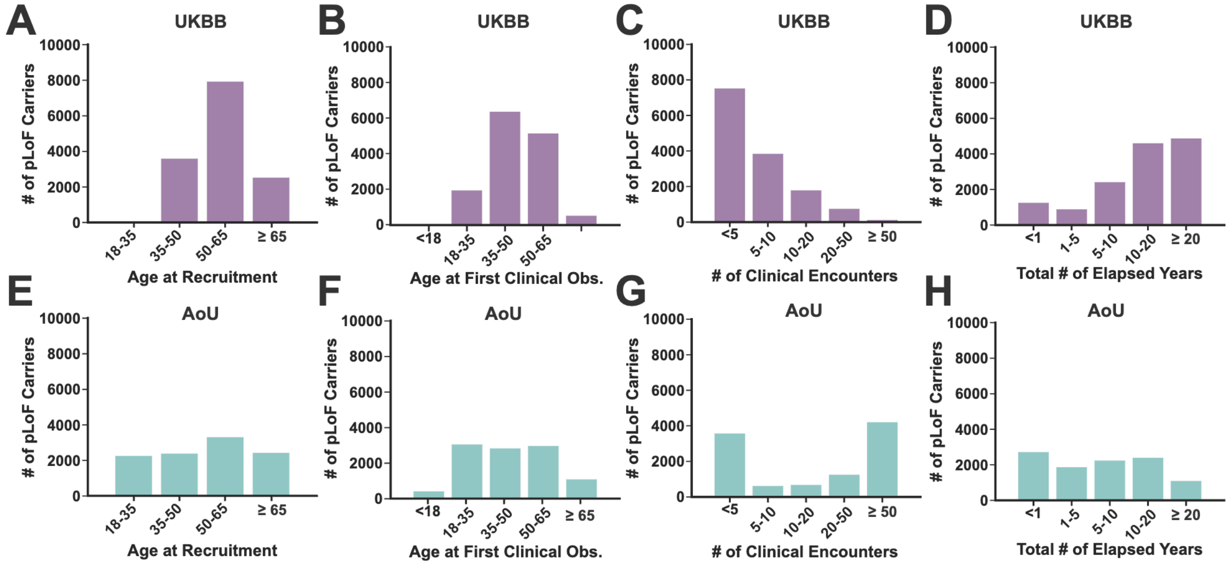

Supplemental Figure 1: Clinical data coverage among pLoF carriers in the UKBB and AoU. (A, E): Distribution over the age at the time of recruitment (A: UKBB; E: AoU). (B, F): Distribution over the age at the first clinical observation (B: UKBB; F: AoU). (C, G): Distribution over the total number of clinical visits (C: UKBB; G: AoU). (D, H): Distribution over the total number of elapsed years between the first and last clinical visits (D: UKBB; H: AoU).

medRxiv preprint doi: https://doi.org/10.1101/2024.09.23.24314008; this version posted October 7, 2024. The copyright holder for this preprint (which was not certified by peer review) is the author/funder, who has granted medRxiv a license to display the preprint in perpetuity.
It is made available under a CC-BY 4.0 International license .

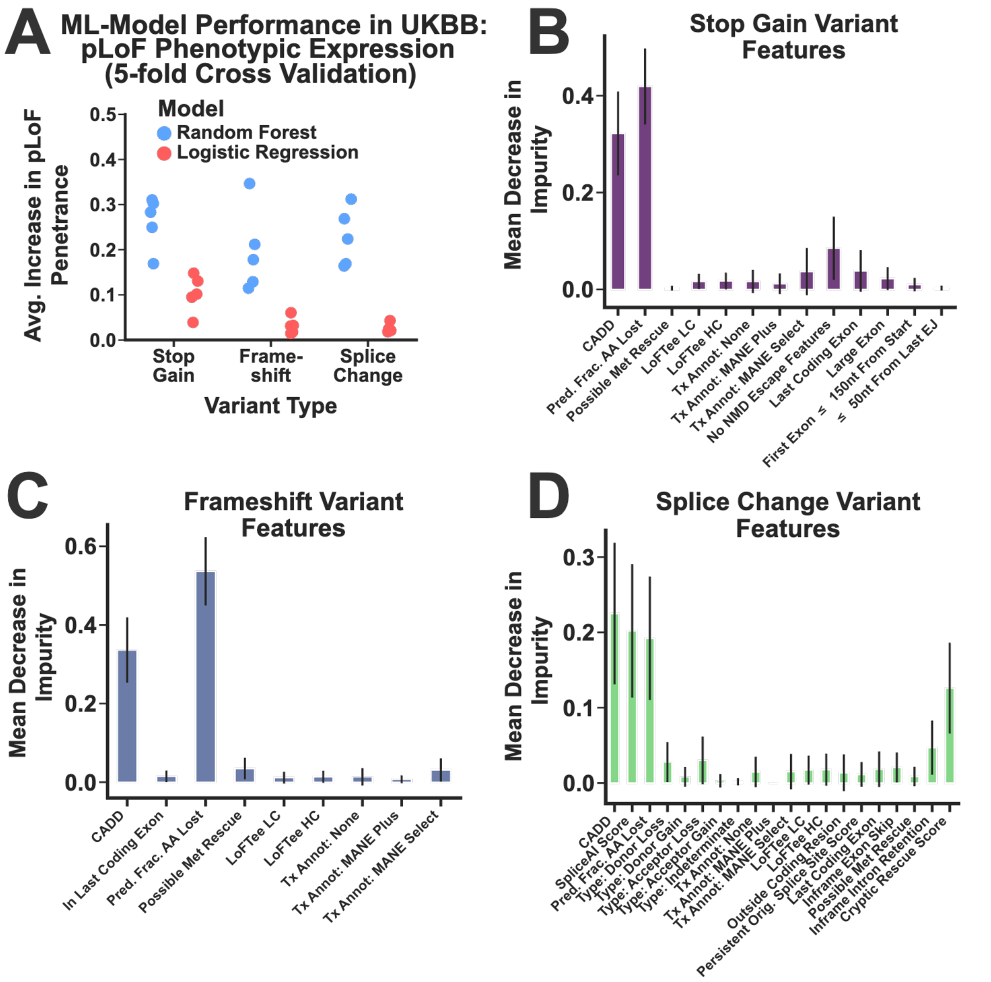

Supplemental Figure 2: Training variant expression prediction models in the UKBB. (A): We evaluated the performance of two different machine learning frameworks for predicting disease expression using pLoF features: Logistic Regression (red) and Random Forests (blue). We assessed performance using the average increase in penetrance achieved by filtering pLoFs according to their expression scores. Random forest models consistently outperformed logistic regression in 5-fold leave-one-out cross validation experiments for all three variant types considered in this study. (B, C, D): In these panels, the variant-intrinsic features used by each model to predict disease expression are displayed on the x-axis. The y-axis displays an approximate measurement of feature importance (the mean decrease in impurity achieved by each pLoF feature). Error bars indicate the standard errors. A: Stop-gain; B: Frameshift; C: Splice Change.
